# The *Salmonella* SPI2 Effector SseI Mediates Long-Term Systemic Infection by Modulating Host Cell Migration

**DOI:** 10.1371/journal.ppat.1000671

**Published:** 2009-11-26

**Authors:** Laura M. McLaughlin, Gregory R. Govoni, Christiane Gerke, Smita Gopinath, Kaitian Peng, Grace Laidlaw, Yueh-Hsiu Chien, Ha-Won Jeong, Zhigang Li, Matthew D. Brown, David B. Sacks, Denise Monack

**Affiliations:** 1 Department of Microbiology and Immunology, Stanford University Medical Center, Stanford, California, United States of America; 2 Department of Pathology, Harvard Medical School, Brigham and Women's Hospital, Boston, Massachusetts, United States of America; Univeristätsklinium Erlangen, Germany

## Abstract

Host-adapted strains of *Salmonella enterica* cause systemic infections and have the ability to persist systemically for long periods of time despite the presence of a robust immune response. Chronically infected hosts are asymptomatic and transmit disease to naïve hosts via fecal shedding of bacteria, thereby serving as a critical reservoir for disease. We show that the bacterial effector protein SseI (also called SrfH), which is translocated into host cells by the *Salmonella* Pathogenicity Island 2 (SPI2) type III secretion system (T3SS), is required for *Salmonella typhimurium* to maintain a long-term chronic systemic infection in mice. SseI inhibits normal cell migration of primary macrophages and dendritic cells (DC) in vitro, and such inhibition requires the host factor IQ motif containing GTPase activating protein 1 (IQGAP1), an important regulator of cell migration. SseI binds directly to IQGAP1 and co-localizes with this factor at the cell periphery. The C-terminal domain of SseI is similar to PMT/ToxA, a bacterial toxin that contains a cysteine residue (C1165) that is critical for activity. Mutation of the corresponding residue in SseI (C178A) eliminates SseI function in vitro and in vivo, but not binding to IQGAP1. In addition, infection with wild-type (WT) *S. typhimurium* suppressed DC migration to the spleen in vivo in an SseI-dependent manner. Correspondingly, examination of spleens from mice infected with WT *S. typhimurium* revealed fewer DC and CD4^+^ T lymphocytes compared to mice infected with *ΔsseI S. typhimurium*. Taken together, our results demonstrate that SseI inhibits normal host cell migration, which ultimately counteracts the ability of the host to clear systemic bacteria.

## Author Summary

Bacteria belonging to the genus *Salmonella* are capable of causing long-term chronic systemic infections, and bacteria primarily reside within macrophages in lymphoid tissues and sporadically are shed in the feces. These persistently infected individuals serve as a significant reservoir for disease transmission. Despite the importance of *Salmonella* as a human pathogen, relatively little is known about the host immune response or virulence mechanisms of long-term systemic infections. Host-adapted *Salmonella* strains invade and manipulate host cells by releasing specialized bacterial effector proteins into the host cell. We show that one of these bacterial effector proteins, SseI (SrfH), is required for *Salmonella* to maintain a long-term chronic systemic infection in mice. SseI is able to block the migration of host immune cells and consequentially attenuate the host's ability to clear systemic bacteria. SseI accomplishes this inhibitory activity in part by associating with the host protein IQGAP1, an important regulator of cell migration. The amino acid sequence of SseI is similar to several other protein sequences of known bacterial pathogens, including PMT/ToxA, a toxin, indicating that these factors may function similarly to one another and may comprise a new family of bacterial effector proteins.

## Introduction

*Salmonella enterica* is a pathogenic bacterial species that is an important cause of disease in humans ranging from gastroenteritis to systemic infections. Host-adapted *Salmonella* serovars disseminate from the gastrointestinal tract and colonize systemic sites. For example, *Salmonella enteric* serovar Typhi (*S. typhi*) causes human typhoid fever, whereas *Salmonella enteric* serovar Typhimurium (*S. typhimurium*) has a broad host range, causing disease in a variety of animals. Strains of *S. typhimurium* cause a typhoid-like disease in mice and usually cause a self-limiting gastroenteritis in healthy human adults. However, *S. typhimurium* can cause systemic infections in humans [1]–[5]. Indeed, recent cases of invasive and recurrent infections in Malawi [3], Mozambique [4], Malaysia [1], and Taiwan [5], were caused by nontyphoidal salmonellae (NTS), which were largely comprised of multidrug-resistant *S. typhimurium* strains [2],[3].

*Salmonella*, a facultative intracellular pathogen, enters the host through the gastrointestinal tract where they preferentially enter microfold (M) cells, which are specialized epithelial cells that sample intestinal antigens and transport them to lymphoid cells in the underlying Peyer's Patches (PP), specialized lymphoid tissue in the small intestine [6],[7]. *S. typhimurium* can also translocate through the intestinal epithelia after uptake by CD-18-expressing immune cells [8]. In order for the infection to extend beyond the intestinal mucosa, *Salmonella* must survive and replicate within macrophages, a privileged niche that allows *Salmonella* to elude the adaptive immune response [9]–[11]. The ability of *Salmonella* bacteria to survive inside of host cells is dependent on the SPI2-encoded T3SS that injects virulence/effector proteins into host cells. Some of the SPI2 T3SS-translocated effector proteins have evolved to allow intracellular bacteria to subvert the bacteriocidal properties of macrophages and to create a specialized *Salmonella*-containing vacuole in which it can replicate [12]. In addition, certain SPI2 secreted effectors can specifically interfere with DC-mediated antigen presentation to CD4^+^ T cells [13]–[15], which are required to control bacterial replication within the host during a long-term systemic *Salmonella* infection [16]. Recently, SPI2 also was implicated in early culling of activated CD4^+^ T cells [17], further illustrating the complex relationship between *Salmonella* and T lymphocytes.

Another important aspect of *Salmonella* pathogenesis is the establishment of an asymptomatic carrier stage that serves as a reservoir of infection as the bacteria are periodically shed and transmitted to new hosts [18]–[20]. Indeed, asymptomatic carriers of *S. typhi* shed the bacilli and are a significant reservoir for this deadly pathogen. To study the basic aspects of host-pathogen interactions during the carrier state, we have characterized a natural model of long-term chronic *Salmonella* infection in mice [21]. This model utilizes a mouse strain that does not typically succumb to infection. *S. typhimurium* can be recovered from systemic sites up to one year after infection and typically these bacteria are sequestered within macrophages in systemic tissues [21],[22].

We previously performed a microarray-based screen to identify *S. typhimurium* factors required for long-term systemic infection in mice [23]. While most SPI2 genes were required for initial colonization of the spleen, the SPI2 effector SseI did not emerge from the screen until 2 weeks post-infection, indicating that SseI plays a role in long-term infection [23]. SseI is a secreted effector that is expressed by intracellular *Salmonella* and translocated across the vacuolar membrane into the host cell cytosol via the SPI2-encoded TTSS [24]. SseI has been shown to bind the actin-crosslinking protein filamin and to co-localize with polymerizing actin in the cytoskeleton and with TRIP6 [25],[26]. The *sseI* gene encodes a 322 amino acid polypeptide whose N-terminal domain is highly similar to several other SPI2 effectors, including SspH2, and this domain is important for translocation and subcellular localization in the host [25]. However, no sequence similarities to the SseI C-terminus have been reported.

In this work, we have demonstrated that SseI is required for maintaining a long-term systemic infection and have defined a mechanism for this function. Specifically, we showed that SseI blocks migration of macrophages and DC in vitro, by a mechanism that involves the interaction of SseI with the host factor IQGAP1, an important regulator of the cytoskeleton and cell migration. *Salmonella* also reduced DC migration in vivo in an SseI-dependent manner, which correlated with a reduction in the number of DC and CD4^+^ T cells in WT *Salmonella*-infected spleens. This data provides evidence for a novel mechanism by which an intracellular pathogen manipulates host cell migration to dampen the ability of the host to clear systemic bacteria.

## Results

### SseI is required for systemic *S. typhimurium* infection in mice

**Figure 1:**
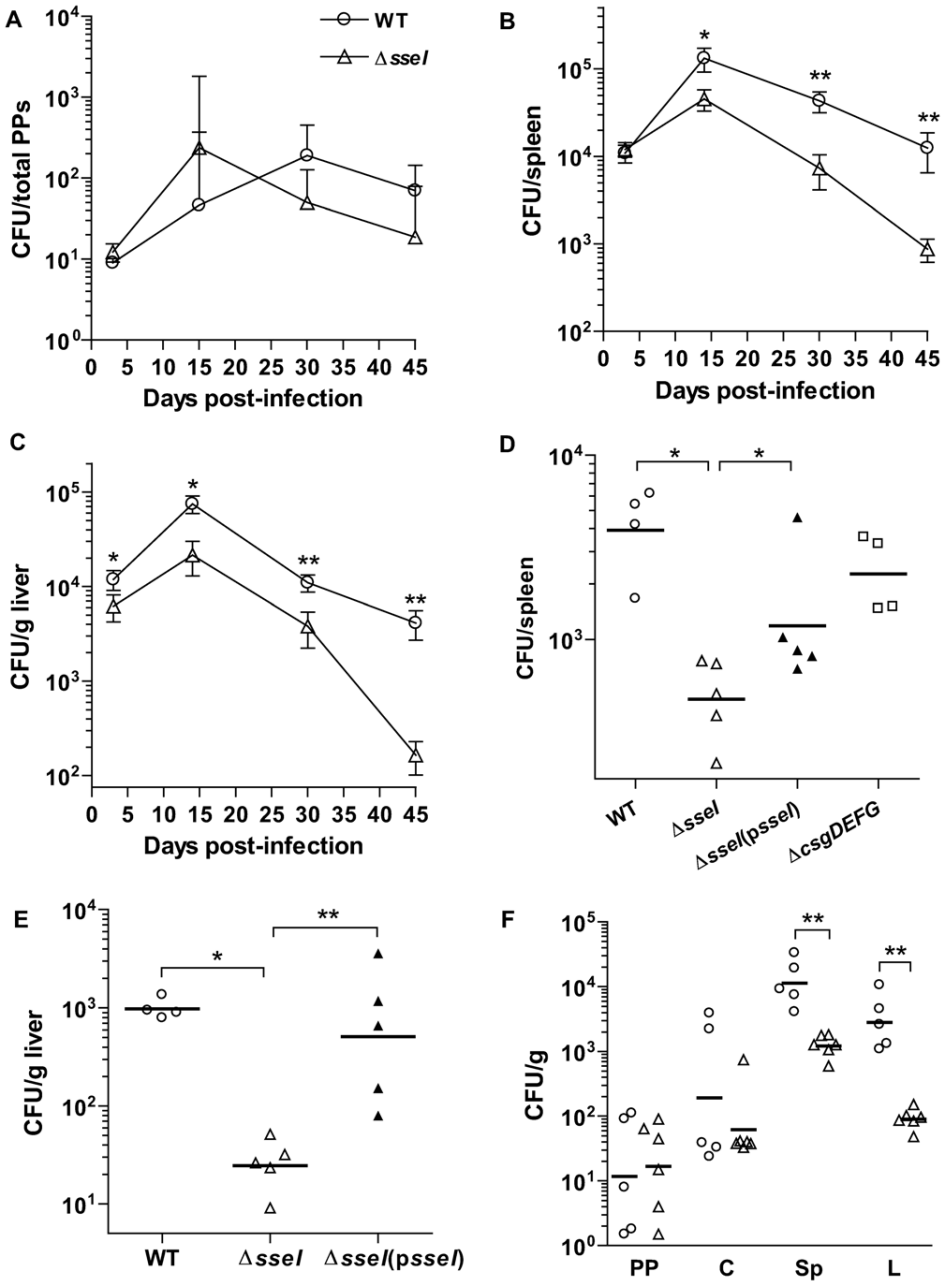
SseI is required to establish a long-term systemic *S. typhimurium* infection in mice. A–C) Mice were infected by IP with WT (circles) or *ΔsseI* (triangles) strains (6.8×10^3^ cfu or 6.6×10^3^ cfu for WT or *ΔsseI*, respectively), and the cfu from the Peyer's Patches (A), spleen (B), and liver (C) are reported. Only positive error bars are shown for Peyer's patches due to their magnitude. Groups of 5 to 8 mice were analyzed/time point, which were 3, 15, 30 and 45d post-infection (p.i.). The experiment was repeated twice. D–E) Mice were infected by IP with WT(p*ACYC184*) (7.3×10^3^ cfu), *ΔsseI*(p*ACYC184*) (6.0×10^3^ cfu), *ΔsseI*(p*sseI*) (5.4×10^3^ cfu, filled triangles), *ΔcsgDEFG*(p*ACYC184*) (5.4×10^3^ cfu, squares) bacterial strains. The cfu from the spleen and liver were determined at 45d p.i. (3–5 mice were analyzed per group). Plasmid retention by 45d p.i. was 29.3±7.0% in the spleen (D) and 22.0±7.0% in the liver (E). F) Mice were orally infected with WT (1.5×10^8^ cfu) or *ΔsseI* (2.06×10^8^ cfu) bacterial strains, and the cfu recovered from Peyer's patches (PP), spleen (Sp), liver, (L), and cecum (C) was measured 34d p.i. (cfu per total PPs shown). The experiment was repeated 3 times; data from a representative experiment is shown. Groups of 5 to 6 mice were analyzed per bacterial strain. *, p<0.05; **, p<0.01, Mann-Whitney U test.

To measure the contribution of SseI to virulence, mice were infected by the intraperitoneal (IP) route with either WT *S. typhimurium* or the *ΔsseI* deletion mutant. The numbers of WT and *ΔsseI* bacteria in the PP, spleen, and liver were measured at 3, 15, 30 and 45d post-infection (Fig. 1A–1C). Although both strains colonized the PP equally well at all time points, the level of WT bacteria was significantly higher than the *ΔsseI* mutant at 30d post-infection in the spleen (5.9-fold more WT than *ΔsseI*) and liver (3.7-fold) (Fig. 1A–1C). In addition, the difference between the WT and *ΔsseI* mutant strains increased between 30d and 45d in the spleen (14.2-fold) and liver (30.6-fold), further demonstrating the importance of SseI to maintaining a long-term infection in these tissues (Fig. 1B and 1C). In contrast, an *S. typhimurium* strain which is deficient for a SPI2 effector that is required for intracellular survival, *sseJ*, (Fig. S1A, S1B and [27]) was attenuated to the same degree at 3d (CI_spleen_ = 0.24±0.07) and 30d (CI_spleen_ = 0.30±0.09) post-infection. To address the possibility that the insertion of a kanamycin resistance gene (*kan^R^*) into the genome contributed to the attenuation of the *ΔsseI* mutant at 45d, we infected mice with another mutant, *ΔcsgDEFG*, that contains a *kan^R^* insertion [23]. The levels of *ΔcsgDEFG* mutant bacteria recovered from systemic tissues were not significantly different from the levels of WT bacteria (Fig. 1D). Thus, the attenuation of the *ΔsseI* mutant in systemic tissues cannot be attributed to minor effects on the growth rate of the bacteria due to the presence of an antibiotic resistance gene over the course of a long-term systemic infection. In addition, a *ΔsseI* strain expressing WT *sseI* in trans (*ΔsseI*(p*sseI*)) was significantly less attenuated at 45d in both the liver (Fig. 1E) and the spleen (Fig. 1D). We also measured the levels of bacteria in PP, cecum, spleen and liver of orally infected mice 34d post-infection. The spleen and liver of WT-infected mice contained significantly higher levels of bacteria compared to *ΔsseI* mutant-infected mice (Fig. 1F), confirming that this SPI2 effector is required to colonize systemic tissues, independent of the route of infection.

### SseI binds directly to the cell migration regulator IQGAP1

**Figure 2:**
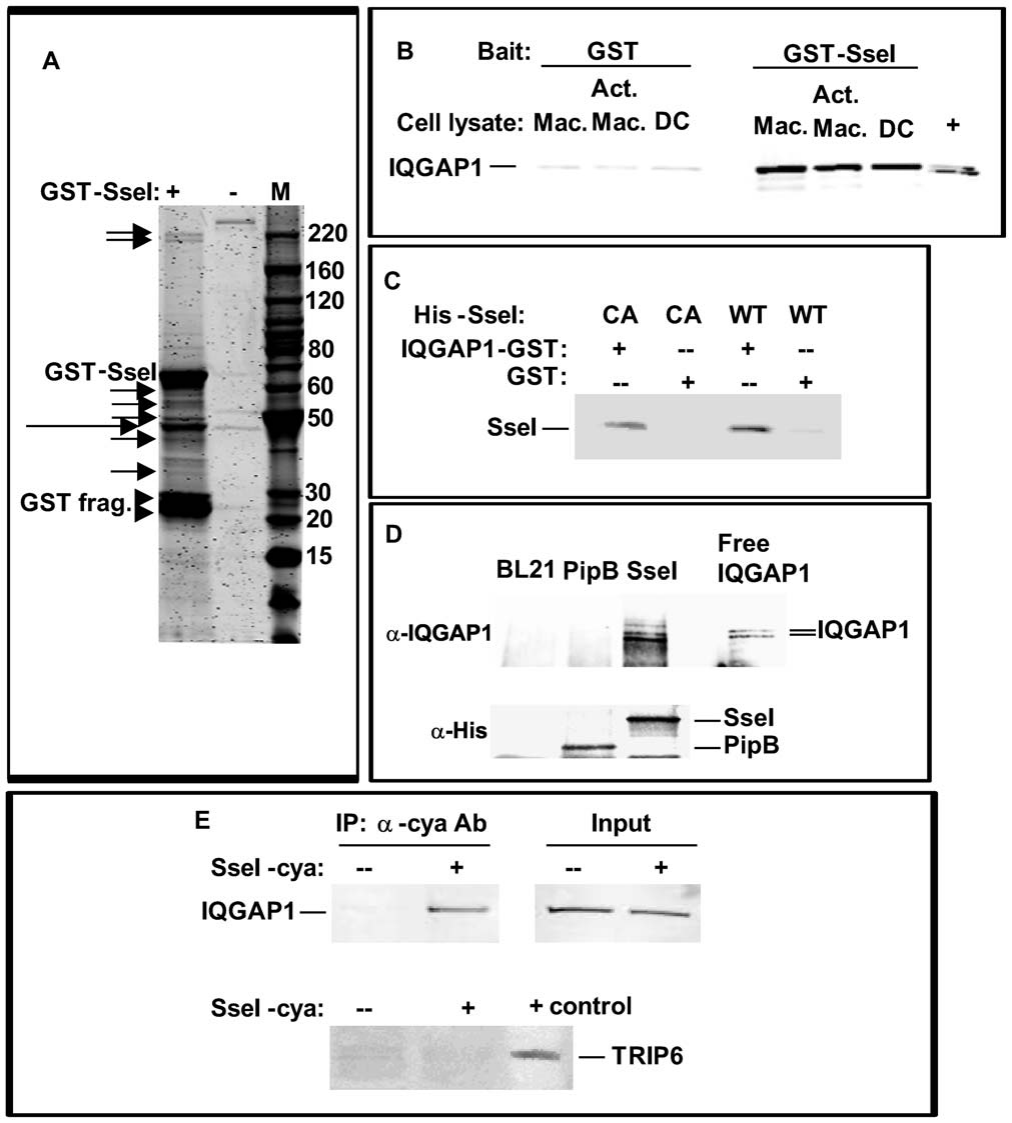
SseI binds directly to the cell migration regulator IQGAP1. A) Purified GST-SseI (or GSH-resin alone) was used to co-precipitate SseI binding proteins from whole cell extracts of BMDM. Bound proteins were eluted and subjected to SDS-PAGE (4–20% gradient gel) and stained with Coomassie blue. Arrows denote bands that were excised and analyzed by mass spectrometry (top doublet is IQGAP1). Long arrow: non-SseI-specific binding; arrowheads: GST-SseI breakdown fragments. B) GST-SseI (or GST alone) pre-bound to GSH-resin was added to whole cell extracts of BMDM (Mac), activated BMDM or BMDC (DC), and bound proteins were immunoblotted for IQGAP1 (+ = HeLa cell extract). C) Purified His-SseI (WT) or His-SseIC178A (CA) proteins were co-precipitated with IQGAP1-GST or GST alone using GSH-resin and bound SseI was detected by immunoblot using anti-His tag antibody. D) In addition, free IQGAP1 was co-precipitated with His-SseI, His-PipB, or resin prepared from *E. coli* BL21 extract alone, and IQGAP1 was detected by immunoblot using anti-IQGAP1 antibody. E) BMDM were infected with *S. typhimurium*-expressing SseI-cya (or SseI alone), and 6h later proteins were immunoprecipitated with anti-CyaA antibody. Bound proteins were immunoblotted for IQGAP1 or TRIP6 (+control = whole cell extract of NIH3T3 cells).

Previous studies have shown that SPI2 and some of the secreted effector proteins are required for intracellular survival and host cell death [9], [28]–[30]. However, we have shown that SseI is not required for bacterial survival in bone marrow-derived macrophages (BMDM) from 129x1/sv J mice or in RAW264.7 macrophage-like cells (Fig. S1A and S1B). SseI also does not regulate host cell death in BMDM (data not shown) or in bone marrow-derived dendritic cells (BMDC) (Fig. S1C and S1D). To determine the molecular targets of SseI, a GST-SseI fusion protein was incubated with primary macrophage lysates, the cell-type in which *Salmonella* is commonly found at systemic sites during long-term infection [20]–[22]. Bound proteins were co-precipitated with GSH-resin, eluted and subjected to SDS-PAGE (Fig. 2A). A band (shown as a doublet in Fig. 2A) migrating at approximately 200 kD was identified by mass spectrometry to be IQGAP1 (21 of 21 tryptic fragments were IQGAP1-specific; no other band was associated with a significant specific protein identity). Immunoblotting analysis showed that GST-SseI specifically co-precipitated IQGAP1 from whole cell extracts made from either BMDM (naïve or activated by pretreatment with 50 ng/ml lipopolysaccharide and 100 U/ml interferon-γ) or BMDC (Fig. 2B).

To determine if SseI can directly bind IQGAP1, co-precipitation studies were conducted with purified proteins (Fig. 2C and 2D). His-tagged SseI (WT) was incubated with GST-IQGAP1 (Fig. 2C) or free IQGAP1 (Fig. 2D), and in both cases SseI and IQGAP1 specifically co-precipitated with one another, indicating that SseI can directly bind IQGAP1 in vitro. IQGAP1 did not co-precipitate with another His-tagged SPI2 secreted effector, PipB [31], indicating that IQGAP1 co-precipitation was specific to SseI (Fig. 2D). To confirm that SseI interacts with IQGAP1 during infection, BMDM were infected with WT bacteria expressing cya-tagged SseI, and whole cell extracts derived from these infected cells were subjected to co-immunoprecipitation using an anti-CyaA antibody (Fig. 2E). IQGAP1 was specifically co-immunoprecipitated with SseI-cya, confirming that these factors interact during *Salmonella* infection. We did not detect an interaction between SseI and TRIP6 in BMDM, in contrast to a previous report using RAW264.7 cells. This may reflect differences in TRIP6 protein levels between these two studies [26].

**Figure 3:**
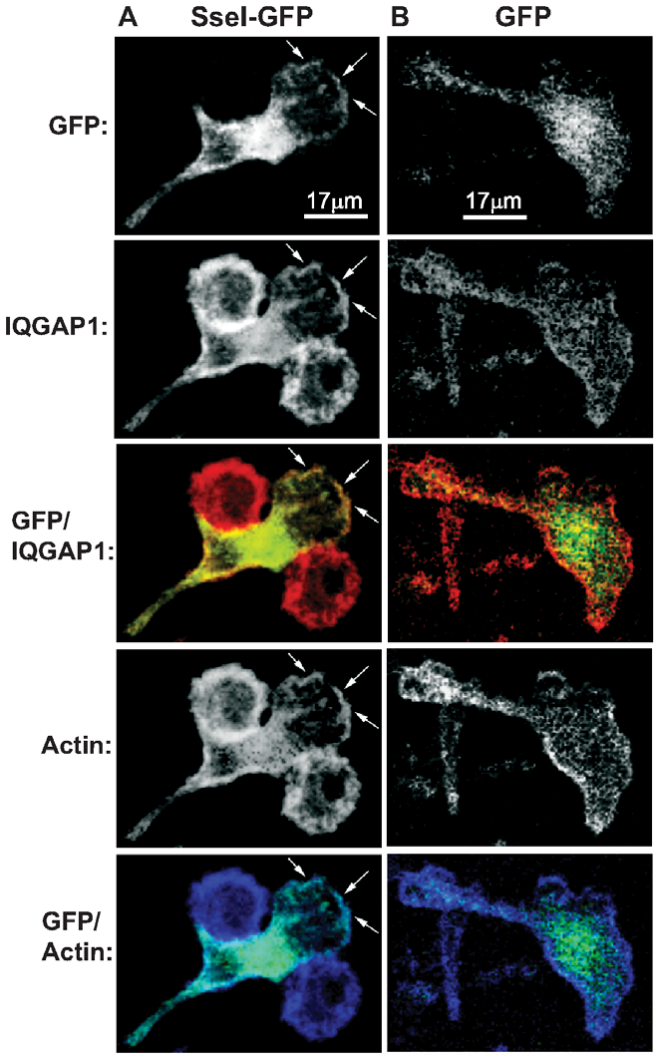
SseI co-localizes with IQGAP1 and actin at the cell periphery. BMDM were transiently transfected with p*sseI-EGFP* (A) or p*EGFP* (B) and then fixed and stained for IQGAP1 (red) and actin (phalloidin, blue). Transfected cells were imaged by confocal microscopy (600×), and the white bars represent 17 microns. Arrows indicate regions of co-localization.

To characterize the nature of co-localization between SseI and IQGAP1 in primary macrophages, BMDM were transiently transfected with an SseI-GFP fusion construct (Fig. 3A) or GFP alone (Fig. 3B). While expression of GFP alone resulted in green fluorescence that was mostly localized to the nucleus with diffuse fluorescence in the cytosol (Fig. 3B), expression of SseI-GFP resulted in an increased concentration of green fluorescence at the cell periphery (Fig. 3A). When these cells were stained for endogenous IQGAP1 (red), significant co-localization between SseI and IQGAP1 was also detectable at the cell periphery, including the lamella (Fig. 3A, Fig. S2A and S2C). BMDM were also stained with phalloidin (blue) to visualize actin within the cytoskeleton [25], and SseI was confirmed to co-localize with polymerized actin (Fig. 3A).

### Infection with *S. typhimurium* causes an irregular pattern of movement in BMDM that is dependent on SseI

**Figure 4:**
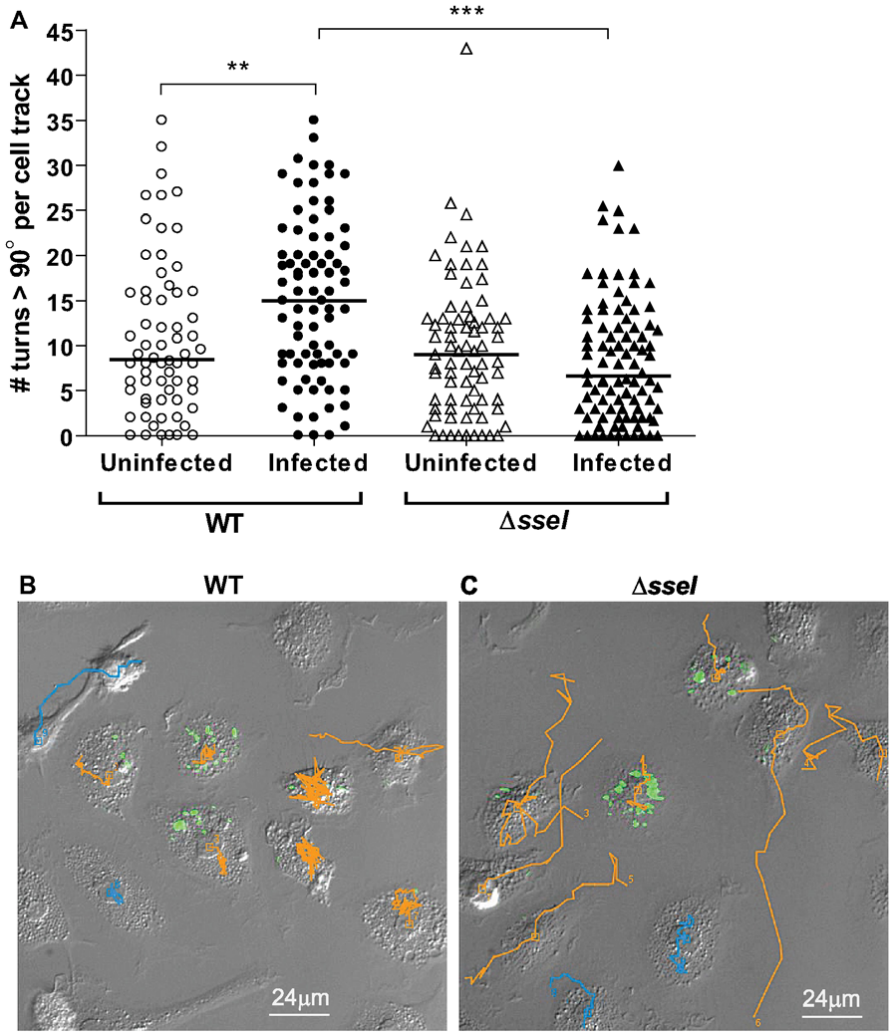
SseI causes *S. typhimurium*-infected BMDM to reverse their direction of travel more frequently. A) BMDM were seeded onto two-chamber glass slides and infected with GFP-expressing strains of WT (WT(p*FPV25.1*)) or *ΔsseI* (*ΔsseI*(p*FPV25.1*)) *S. typhimurium* for 24h. Four locations from each chamber were imaged by time-lapse microscopy (DIC and fluorescence; images were taken every 3min; 45 images were taken in all per movie). The number of times a cell changed its direction of movement more than 90° (per video) are reported for infected cells and their uninfected neighbors (bars represent the median, the data are compiled from 28 total movies (14 movies per bacterial strain) performed in 4 independent experiments, n = 66 for uninfected cells (circles) and n = 82 for infected cells for WT *S. typhimurium*-infected BMDM (filled circles), n = 75 for uninfected cells (triangles) and n = 96 for infected cells for *ΔsseI S. typhimurium*-infected BMDM (filled triangles)). **, p-value<0.01 and ***, p-value<0.001; Mann-Whitney U test. B–C) The frames and cell-tracks of two representative movies are shown (B, WT; C, *ΔsseI*, tracks of uninfected cells are shown in blue and those of infected cells are orange), and the full videos are available online (Videos S1 and S2).

IQGAP1 is a 190 kD scaffolding protein that binds actin and regulates the cytoskeleton and cell migration machinery [32]. Since SseI binds to IQGAP1, we tested the hypothesis that SseI interferes with cell motility. BMDM were seeded onto two-chamber slides, and each chamber was infected with either WT or *ΔsseI* strains of *S. typhimurium* expressing GFP. Individual infected cells (as well as their uninfected neighbors) were monitored by time-lapse microscopy at 24 h p.i.. The cells were tracked and analyzed for the number of times each cell reversed its direction of movement (>90° turn between 3 consecutive frames was scored as a reversal) and for their net displacement. Surprisingly, cells infected with WT bacteria made significantly more turns that were >90° compared to cells infected with *ΔsseI* mutant bacteria (Fig. 4A; p<0.001). In addition, WT-infected cells made significantly more turns compared to their uninfected neighbors (Fig. 4A; p<0.01), indicating that the intracellular bacteria modulated the cells' normal patterns of movement. These results also demonstrate that the intracellular bacteria exert their influence specifically on the infected cell and that the modulation of cell movement is not due to bystander effects. The cell tracks of two representative movies of WT- (Fig. 4B, Video S1) and *ΔsseI*- (Fig. 4C, Video S2) infected BMDM are shown. The median net displacement did not change significantly with infection (data not shown). Taken together, our data demonstrate that *S. typhimurium* alters the movement of infected phagocytic cells in a cell autonomous fashion by an SseI-dependent mechanism.

### SseI inhibits the directed migration of BMDM and BMDC

**Figure 5:**
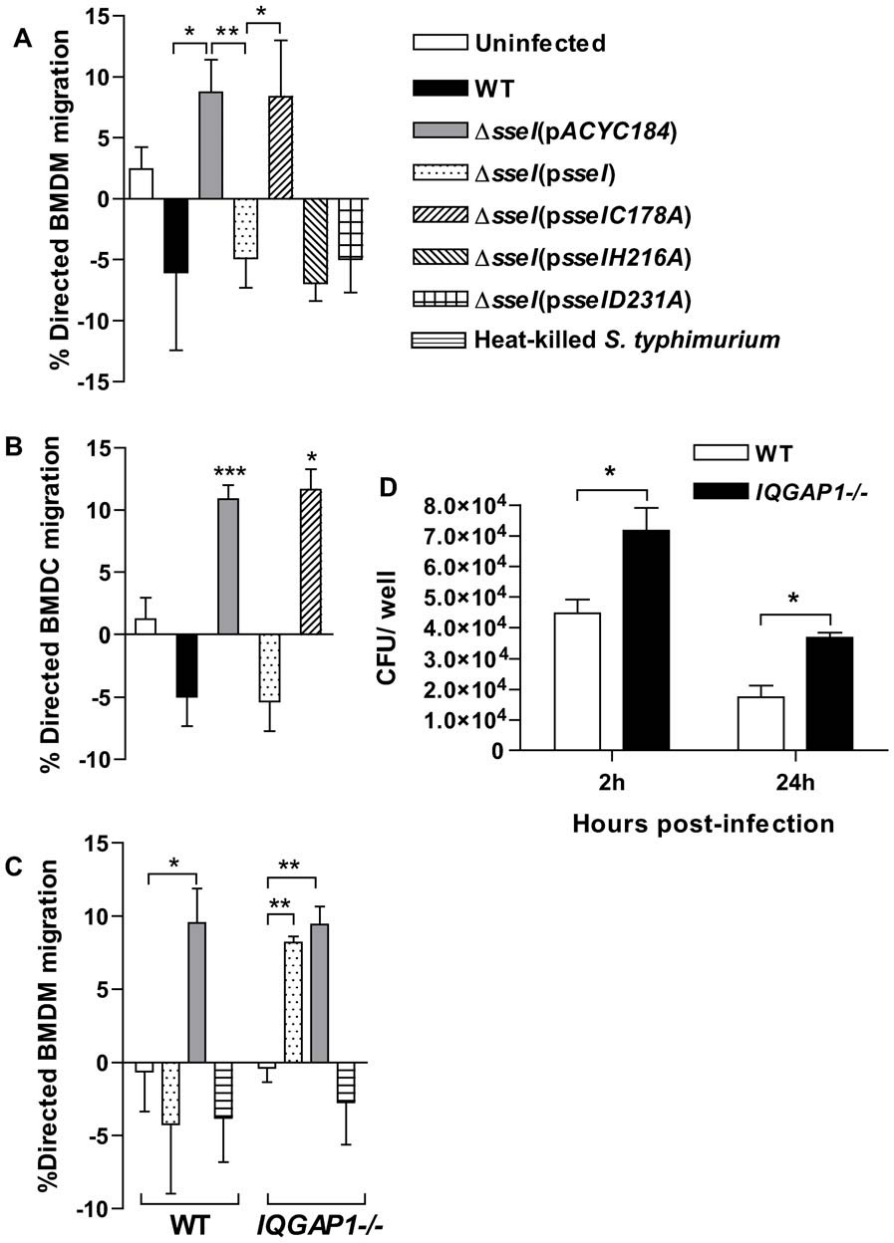
SseI inhibits directed migration of BMDM and BMDC in an IQGAP1-dependent manner. A) BMDM seeded on transwell filters were infected with the indicated strain of *S. typhimurium*, and at 24h, heat-killed *Salmonella* (equivalent of 0 or 12.5 million cfu) was added to the baso-lateral compartment as an attractant. The percentage of cells migrating through the filter was measured 5h later by confocal microscopy. The % migration was calculated: (the % migration of BMDM receiving the heat-killed *Salmonella*) – (% BMDM migration without the attractant) = % directed BMDM migration. The results are presented as the average of 5 independent experiments; *, p<0.05 and **, p<0.01 when comparing in a two-sample Student's t tests. B) BMDC were seeded and infected as in (A), and then 100ng/ml CCL-19 was used as the chemoattractant to measure the % directed migration (calculated as in (A)). The mean and SEM were calculated from at least 3 replicates. The data are representative of two independent experiments; *, p<0.05 when comparing the % directed BMDC migration to zero in a one-sample Student's t test. C) BMDM from age-matched WT and *IQGAP1^−/−^* mice were treated as in (A) and the results are the average of three independent experiments (*, p<0.05 and **, p<0.01 when comparing in a two-sample Student's t test). D) The amount of WT *S. typhimurium* protected from gentamicin in WT and *IQGAP1^−/−^* BMDM was measured 2h and 24h after infection and is reported as the average of the total cfu per well; *, p<0.05 when comparing WT to *IQGAP1^−/−^* in a two-sample Student's t test. There was no significant difference in the amount of bacteria protected from gentimicin in BMDM when comparing WT, *ΔsseI*, and *ΔsseI*(p*sseI*) *S. typhimurium* strains (Fig. S1 and data not shown).

Since our results demonstrated that SseI interferes with normal cell movement (Fig. 4) and that SseI binds IQGAP1 (Fig. 2), a host protein that promotes cell migration, we next tested whether SseI influenced the directed migration of primary BMDM and BMDC. These primary cells were seeded onto transwells and infected with WT or mutant bacterial strains at an MOI of 10∶1, conditions that resulted in 22±2% of the cells infected. The percentage of host cells that migrated specifically toward an attractant was quantified by confocal microscopy. BMDM infected with WT bacteria did not migrate toward the attractant (Fig. 5A). As Videos S1 and S2 show, BMDM are highly motile cells, and for all samples, there was a low basal level of migration to the bottom of the filter in the absence of attractant. In the case of SseI-expressing bacteria, this basal level of migration was slightly higher than when the attractant was added, resulting in the negative values (Fig. 5A). However, these negative values were not significantly different from zero when tested in one-sample Student's t test. In contrast to WT *Salmonella*, BMDM infected with *ΔsseI* mutant bacteria readily migrated toward the attractant (Fig. 5A). Furthermore, the ability of the *ΔsseI* mutant strain to inhibit host cell migration was fully restored by adding back a WT copy of the *sseI* gene, confirming a specific role for SseI in the inhibition of directed migration (Fig. 5A and 5B). In tissue, mature DC migrate towards the CCR7 ligand, CCL19, in order to present antigen to T cells in secondary lymphoid tissue [33]. Similar to our results with BMDM, BMDC infected with WT bacteria did not migrate toward CCL19, whereas BMDC infected with the *ΔsseI* strain did (Fig. 5B). Inhibition of BMDC migration was not due to any alteration in CCR7 surface levels, as WT- and *ΔsseI*-infected BMDC expressed the same levels of CCR7 (Fig. S5G). Thus, *S. typhimurium* interferes with the directed migration of host phagocytic cells via a novel mechanism that depends on the secreted bacterial effector SseI.

### SseI-mediated inhibition of migration is dependent on IQGAP1

While IQGAP1 promotes cell migration [34], it is not absolutely required (Fig. S3). Therefore, we examined the role of IQGAP1 in SseI-dependent inhibition of directed migration. To test whether IQGAP1 is required for this SseI-dependent activity, the ability of SseI to regulate host cell migration was compared in BMDM derived from WT and *IQGAP1^−/−^* mice. As expected, WT BMDM infected with the complemented *sseI* mutant bacterial strain (*ΔsseI*(p*sseI*)) did not migrate toward heat-killed *Salmonella* (Fig. 5C). In contrast, *IQGAP1^−/−^* BMDM infected with the complemented *sseI* mutant bacterial strain migrated toward the attractant, and the levels of migration were similar to BMDM infected with the *ΔsseI* mutant (Fig. 5C), indicating that IQGAP1 is necessary for SseI-dependent regulation of cell migration. Another possible explanation is that there was decreased bacterial uptake by the *IQGAP1^−/−^* BMDM [35],[36]; however, the intracellular bacterial loads in *IQGAP1^−/−^* BMDM were not less than those in WT BMDM (Fig. 5D). These data confirm that the loss of the ability of *S. typhimurium* to inhibit migration of *IQGAP1^−/−^* BMDM was not due to decreased intracellular bacterial numbers (Fig. 5D). Furthermore, we demonstrated that *S. typhimurium* infection induced IQGAP1-independent pathways of cell migration, which was dependent on infection with intact bacteria (as infection with heat-killed bacteria did not induce migration, Fig. 5C). Thus, infection with intact bacteria induced migration that was independent of IQGAP1 and SseI. However, the concomitant presence of both the bacterial effector, SseI, and the host factor, IQGAP1, resulted in a dominant interference with host cell migration.

### SseI plays a distinct role in cell adhesion when expressed in RAW264.7 cells

**Figure 6:**
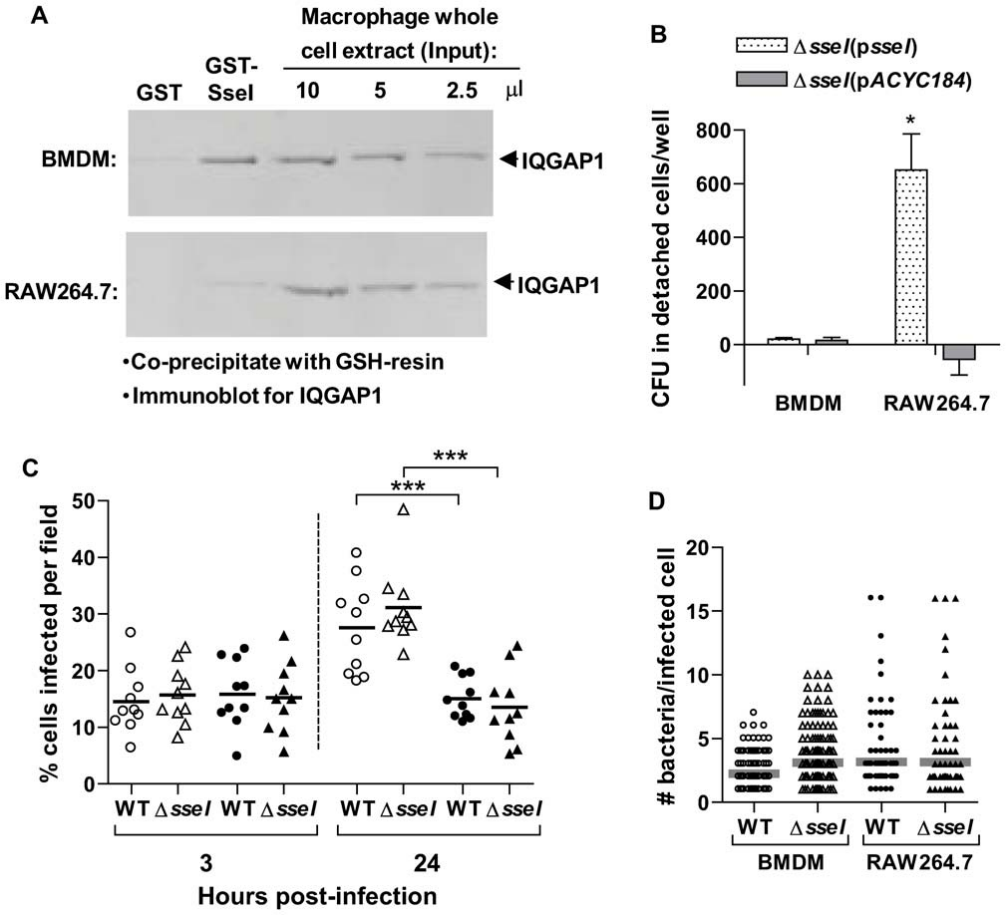
In RAW264.7 cells, *S. typhimurium* mediates SseI-dependent detachment, but SseI does not bind IQGAP1. A) As in Figure 2B, GST or GST-SseI was combined with whole cell extracts of either WT BMDM or RAW264.7 cells and co-precipitated with GSH-resin. Bound proteins, as well as the indicated amounts of the original whole cell extracts, were subjected to SDS-PAGE and immunoblot detection of IQGAP1. B) WT BMDM and RAW264.7 cells in 6-well plates (5×10^5^ cells/well) were infected with the indicated *S. typhimurium* strain. After infection, media with or without heat-killed *Salmonella* was added to the cells, and 24h later, the cells that had detached were harvested, lysed and plated for cfu. The data are presented as the difference between the cfu recovered from cells treated with heat-killed *Salmonella* and that of untreated cells. The data are representative of 3 independent experiments (each in triplicate); *, p<0.05 in a two-sample Student's t test when comparing WT-infected cells to that of *ΔsseI*. C–D) BMDM and RAW264.7 cells were seeded onto coverslips and then treated as in part B. The cells were then fixed and stained for *S. typhimurium* and actin (phalloidin). The % of cells infected (C, 3h and 24h p.i.) and the number of bacteria per infected cell (D, 24h p.i.) were quantified by confocal microscopy. Ten fields (averaging 40 cells/field) were counted per sample group; bars represent the geometric means. ***, p<0.001 when comparing in a two-sample Student's t test.

A previous report published by Worley et al. [26] demonstrated that RAW264.7 cells (a transformed monocytic-like cell line) expressing SseI moved through and detached from transwells at an increased rate, and that this activity was dependent on the host factor TRIP6 [26]. However, in our experiments we were unable to detect an interaction between TRIP6 and SseI in primary macrophages (Fig. 2E). Furthermore, GST-SseI-co-precipitation of IQGAP1 in RAW264.7 cells is dramatically reduced even though IQGAP1 is present (Fig. 6A), perhaps indicating that the binding site is blocked. Thus, the fact that SseI interacted with different host factors in RAW264.7 cells as compared to primary BMDM and BMDC suggested that SseI may function differently in the RAW264.7 cell line.

In the migration assay of Worley et al. [26], detection of migration relied on cells migrating through and detaching from the transwell (measuring both migration and loss of adherence simultaneously), whereas in our study migration was scored by counting cells that traversed the transwell without detaching (specifically measuring directed migration). To measure the effect of SseI on cell adherence, we compared the levels of BMDM and RAW264.7 cells that had detached from tissue culture plates when infected with either the complemented *sseI* mutant bacterial strain (*ΔsseI*(p*sseI*)) or the *sseI* mutant bacterial strain containing the empty vector (*ΔsseI*(p*ACYC184*)). Since the levels of host cell detachment were very low, we counted the number of bacteria that were released into the supernatant as described by Worley et al. [26]. Infection of RAW264.7 cells with the complemented *sseI* mutant bacterial strain (Fig. 6B) or WT strain (data not shown) resulted in significantly higher numbers of bacteria released into the supernatant compared to background levels. Thus, we observed an SseI-dependent detachment in RAW264.7 cells, but not in BMDM, suggesting that SseI regulates cell adherence in RAW264.7 cells but not in primary macrophages. As expected, the percentages of RAW264.7 cells infected with either the WT or *sseI* mutant bacterial strain were not significantly different at 3h or 24h p.i. (Fig. 6C), demonstrating that SseI-dependent detachment of infected RAW264.7 cells at 24h could not be due to an SseI-dependent difference in the percentage of infected cells. Furthermore, there was not a significant difference in the average number of WT or *sseI* mutant bacteria per RAW264.7 cell at 24 h (Fig. 6D), which is consistent with our results when comparing intracellular bacterial replication in a gentamicin protection assay (Fig. S1A and S1B). Thus, the SseI-dependent detachment of infected RAW264.7 cells could not be attributed to SseI-dependent differences in intracellular bacterial growth. While the average number of bacteria per infected cell was not significantly different between BMDM and RAW264.7 cells, there were more RAW264.7 cells infected with >10 bacteria at 24h than BMDM (Fig. 6D). Thus, it is possible that SseI-dependent detachment is not detectable in BMDM due to the lack of cells with very high numbers of bacteria and is another possible explanation for any differences between results obtained with RAW264.7 cells [26] compared to BMDM.

### Cysteine 178 is critical for SseI-dependent inhibition of migration and for colonization of host systemic tissues

**Figure 7:**
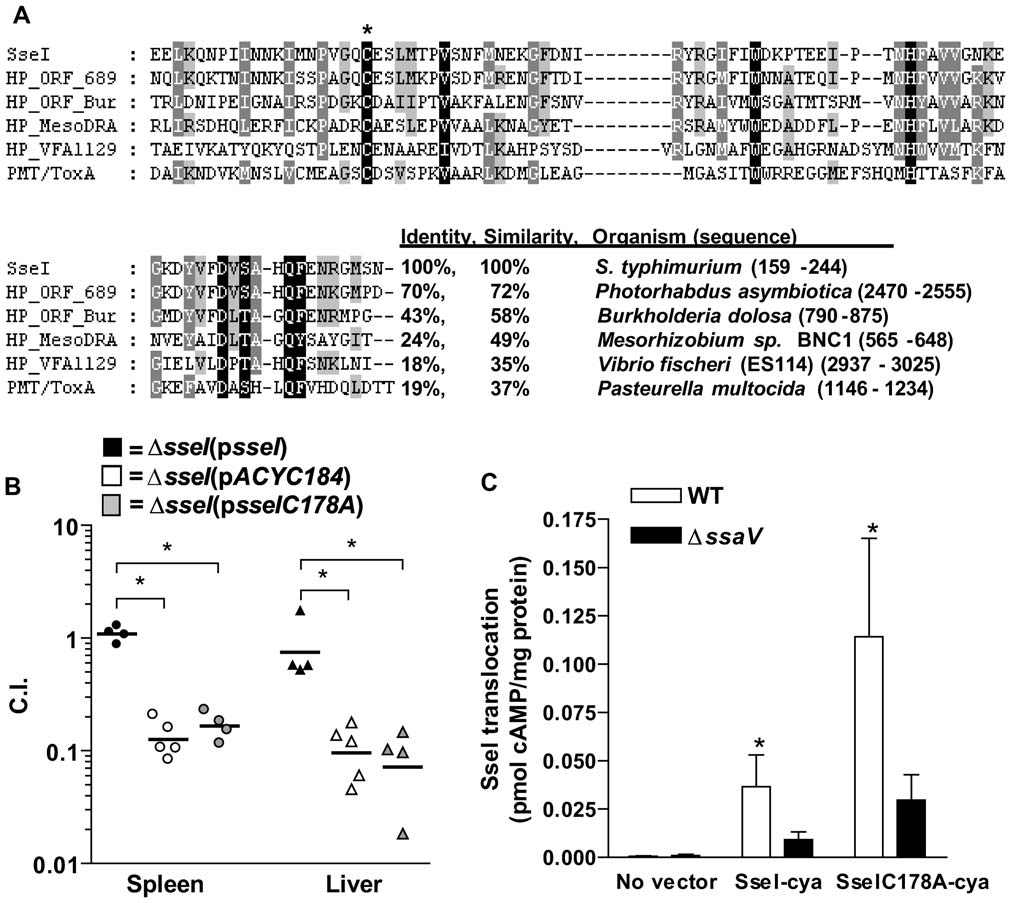
Cysteine 178 is critical for SseI function in vivo. A) Amino acid sequences with similarity to the C-terminal domain of SseI (159–244) are shown. Conserved residues are highlighted and C178 of SseI is starred. B) Mice were infected (IP) with equal amounts (4×10^3^ cfu) of WT and *ΔsseI* transformed with p*sseI*, p*sseIC178A*, or p*ACYC184* (empty vector), and the competitive index was measured 2 weeks p.i.. Groups of 4 to 5 mice were analyzed per condition: *, p<0.05 in the Mann-Whitney U test. C) WT and *ΔssaV* (SPI2 mutant) strains were transformed with p*sseI-cya* or p*sseIC178A-cya* and used to infect RAW264.7 macrophages; the resulting adenylate cyclase activity in macrophage cytosolic fractions was measured at 6h p.i. and is expressed as pmol cAMP/µg protein. *, p<0.05 when comparing WT and *ΔssaV* in a two-sample Student's t test, and the data are presented as the average of three independent experiments.

To investigate the molecular mechanism of SseI action, the C-terminal domain (159–322) of SseI was subjected to a position iterative (PSI-) BLAST search, which uncovered sequence similarity to several hypothetical proteins, as well as to the bacterial toxin PMT/ToxA (Fig. 7A). Three of the aligned sequences are from known insect and mammalian pathogens (*P. asymbiotica* [37], *B. dolosa* [38], and *P. multocida* [39]), suggesting that these genes may comprise a family of bacterial virulence factors. PMT/ToxA, recently shown to be a deamidase [40], is required for virulence [39] and has been shown to inhibit DC migration and impair actin reorganization; all these activities have been shown to be dependent on a critical cysteine residue at position 1165 [41].

To test whether the corresponding residue in SseI (C178) was required for its function, the SseIC178A point mutant protein was constructed and compared to WT SseI protein. Co-precipitation studies showed that the SseIC178A mutant protein had similar binding affinity for IQGAP1 as compared to WT SseI (Fig. 2C and S4). However, the *S. typhimurium* strain expressing SseIC178A (*ΔsseI*(p*sseIC178A*) did not inhibit BMDM migration, similar to the *ΔsseI* strain (Fig. 5A). In contrast, mutation of the conserved H and D residues in SseI (H216A and D231A) did not interfere with SseI-dependent regulation of migration (Fig. 5A). To test whether C178 is also critical to SseI function in vivo, the virulence of the *ΔsseI*(p*sseIC178A*) mutant strain was compared to WT in mixed infections where mice were infected with a 1∶1 ratio of WT *S. typhimurium* and *ΔsseI S. typhimurium* strains transformed with p*sseI*, p*sseIC178A*, or p*ACYC184* (empty vector) (Fig. 7B). The WT strain out-competed the *ΔsseI*(p*sseIC178A*) strain to the same extent as the *ΔsseI*(p*ACYC184*) strain, demonstrating that C178 is critical for SseI function in vivo (Fig. 7B). The SseIC178A mutant protein was expressed and translocated through the SPI2 T3SS at levels comparable to WT SseI (Fig. 7C), confirming that loss of activity was due to specific mutation of C178 [42].

### SseI-dependent suppression of DC migration in vivo correlates with lower numbers of DC and CD4^+^ T cells in infected spleens

**Figure 8:**
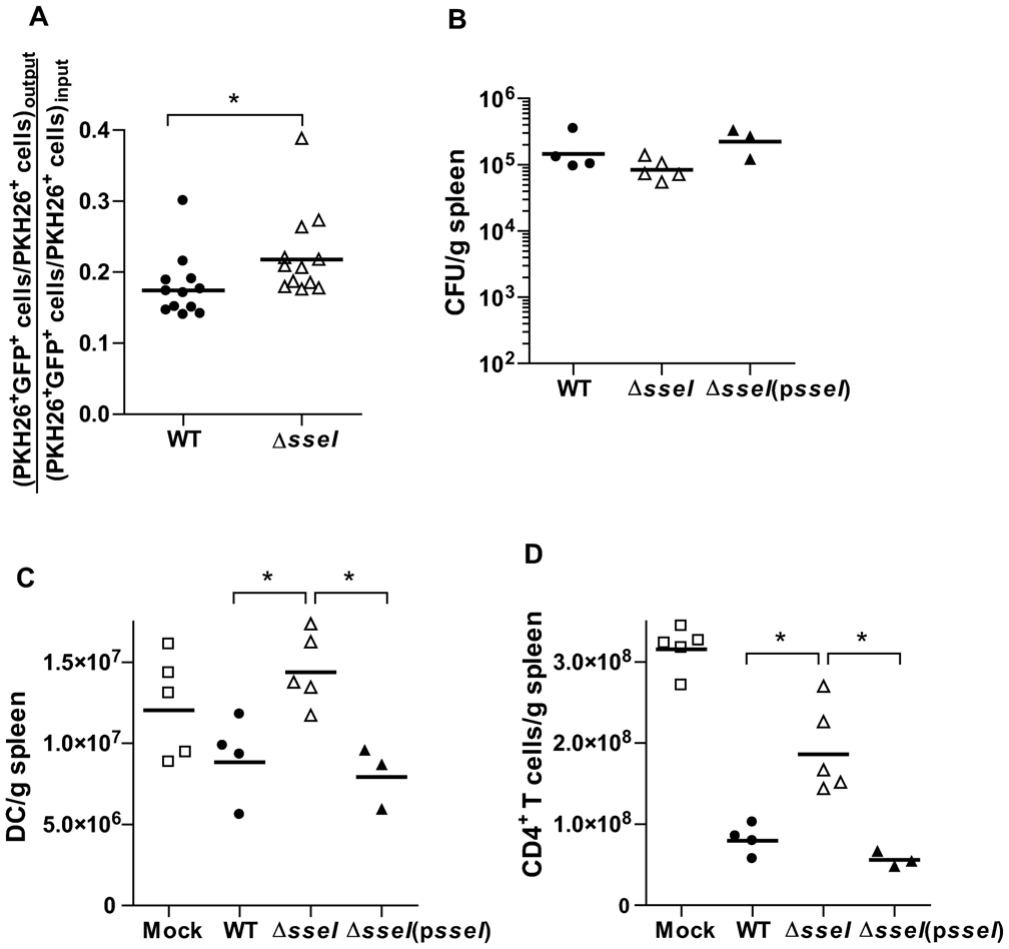
SseI-dependent suppression of DC migration in vivo correlates with lower numbers of DC and CD4^+^ T cells in the spleen of mice infected with WT *S. typhimurium*. A) BMDC stained with the vital dye PKH26 and infected with GFP-expressing strains of WT (WT(p*FPV25.1*) or *ΔsseI* (*ΔsseI*(p*FPV25.1*)) *S. typhimurium* (chased with 100µg/ml gentamicin to kill remaining extracellular bacteria) were injected into 129x1/sv J mice at 5 million cells per mouse. Single cell suspensions were prepared from spleens and analyzed by FACS to detect PKH26 and GFP signals in BMDC that had migrated to the spleen at 6h post-injection. The PKH26-labeld BMDC that were infected with GFP^+^ *S. typhimurium* ex vivo were also analyzed by FACS to determine input values. The results are expressed as the in vivo migration index = (#PKH26^+^GFP^+^ cells/ #PKH26^+^GFP^−^ cells)_output_/(#PKH26^+^GFP^+^ cells/ #PKH26^+^GFP^−^ cells)_input_. *, p<0.05; Mann-Whitney U test. B–D) The bacterial loads (B) and the cellular composition (C–D) of the spleens of 129x1/sv J mice infected (IP) with WT, *ΔsseI* or *ΔsseI*(p*sseI*) *S. typhimurium* strains was analyzed at12d p.i. by plating for cfu (B) or by FACS (C–D) for the numbers of DC (C) and CD4^+^ T cells (D) per spleen (Mock = uninfected). *, p<0.05; Mann-Whitney U test. The mean and SEM were calculated from 3–5 replicates per sample. Data from a representative experiment are shown. The experiment was repeated 3 times with similar results.

We found that WT *S. typhimurium* inhibits migration of infected BMDC in vitro by a mechanism that depends on SseI. Therefore, we investigated the potential role of SseI in inhibiting migration of *Salmonella*-infected DC in vivo (Fig. 8A). BMDC stained with the vital dye PKH26 were infected with GFP-expressing WT(p*FPV25.1*) or *ΔsseI*(p*FPV25.1*) strains of *S. typhimurium*, and approximately 5×10^6^ labeled BMDC (∼50% GFP^+^) were injected into 129x1/sv J mice by the IP route. The migration of the injected BMDC to the spleen was measured at 6h post-injection by flow cytometry. To control for heterogeneity in the exact numbers of migrating BMDM between mice, we calculated an in vivo migration index for each injected animal. We defined the in vivo migration index as the ratio of infected to uninfected BMDC (GFP^+^ PKH26^+^ cells/GFP^−^ PKH26^+^ cells) that had migrated to the spleen (output) divided by the ratio of the infected to uninfected BMDC (GFP^+^ PKH26^+^ cells/GFP^−^ PKH26^+^ cells) used for injection (input) (details in Materials and Methods). An in vivo migration index value of less than 1 would indicate that infection with *S. typhimurium* attenuates the migration of BMDC to the spleen. By comparing the migration indices for WT- and *ΔsseI* mutant-infected cells, we could assess the relative contribution of SseI to the modulation of host cell migration in vivo. Indeed, the migration index obtained with *ΔsseI*-infected BMDC was significantly higher (25% increase) than the migration index obtained with WT-infected BMDC (Fig. 8A; p<0.05). This modest 25% difference between the in vivo migration indices obtained from the WT and *ΔsseI S. typhimurium* strains (just 6h post-injection) also is consistent with the gradual attenuation of the *ΔsseI* mutant in systemic tissues over a period of 1.5 months.

In addition, the cellular composition of WT(p*ACYC184*)-, *ΔsseI*(p*ACYC184*)-, or *ΔsseI*(p*sseI*)-infected spleens were compared at 12d post-infection when the numbers of WT and mutant strains of bacteria in the spleens were not significantly different (Fig. 8B). While the numbers of GR-1^+^ cells were not significantly different between the WT and mutant strains (Fig. S5A), the numbers of DC and CD4^+^ T cells in the spleens of *ΔsseI*(p*ACYC184*)-infected mice were significantly higher than those of WT(p*ACYC184*)- and *ΔsseI*(p*sseI*)-infected mice (Fig. 8C and 8D), suggesting a more pronounced T cell response in the *ΔsseI*-infected mice. These data are in accordance with previous results showing that *Salmonella* interferes with T cell proliferation in vivo and inhibits DC-mediated antigen presentation by a SPI2-dependent mechanism [13],[15]. However, we (Fig. S5B, S5C, S5D) and others [14] have shown that SseI does not directly interfere with DC-antigen presentation to T cells in vitro. Furthermore, surface upregulation of MHC-II and B7.2 in *Salmonella*-infected DC, which is not altered in a SPI2-dependent manner [13],[15], were the same in WT and *ΔsseI* infections in vivo and in vitro (Fig. S5E, S5F, S5H). While it is unlikely that SseI directly modulates the CD4^+^ T cell response, our data demonstrated that SseI suppressed DC migration in vivo, which correlated with the ability of *Salmonella* to continuously maintain a systemic infection for at least 45d.

## Discussion

SseI is continuously required for *S. typhimurium* to colonize the spleen and liver and to maintain a long-term systemic infection, as the attenuation of *ΔsseI* mutant strains significantly increased over the duration of infection (Fig. 1B and 1C). In contrast, SseI does not contribute to colonization of Peyer's patches and cecum within the GI tract (Fig. 1A and 1F). This tissue-specificity may reflect differences in *S. typhimurium* localization (i.e. extracellular vs. intracellular), host cell interactions or host immune clearance mechanisms. This is a question that we are currently investigating.

SseI specifically binds the cell migration regulator IQGAP1 (Fig. 2 and Fig. 3) and inhibits migration of BMDM and BMDC toward known attractants (Fig. 5A and 5B). IQGAP1 is a large scaffolding protein that binds actin and several small G proteins, including those in the Rho GTPase family, such as Cdc42 and Rac1, but does not bind RhoA itself [34],[43],[44]. However, all of these Rho family GTPases play important roles in cell migration [45]. IQGAP1 binding inhibits the intrinsic GTPase activity of Cdc42 and Rac1 and prolongs G protein signaling [44],[46]. IQGAP1 also captures microtubules (via CLIP-170 and APC), thereby regulating the directionality of cell migration [47],[48]. Cdc42 and Rac1 are important regulators of IQGAP1 activity, because overexpression of IQGAP1 mutants that cannot bind Cdc42 or Rac1 induce the formation of multiple leading edges and inhibit cell migration in a dominant manner [34],[47]. We have shown that *Salmonella*-infected macrophages exhibit a higher frequency of reversals in their direction of movement and that this change in movement behavior is dependent on SseI (Fig. 4A). SseI directly binds IQGAP1 (Fig. 2C and 2D) in primary macrophage and DC lysates (Fig. 2B) and during *Salmonella*-infection of primary macrophages (Fig. 2E). We have shown that while infection with live bacteria that are lacking *sseI* induced macrophage migration, infection with SseI-expressing bacteria blocked directed migration in an IQGAP1-dependent manner (Fig. 5C), demonstrating a functional interaction between SseI and IQGAP1. Whether SseI interferes with the regulation of IQGAP1 or causes IQGAP1 to adopt an aberrant activity remains to be determined. Ultimately however, this interaction between SseI and IQGAP1 leads to the interference in the host cell's ability to efficiently migrate toward an attractant. The determination of the role of *IQGAP1* in the ability of *S. typhimurium* to cause long-term systemic infection awaits the generation of *IQGAP1^−/^* ^−^ 129x1/sv J mice.

The C-terminal sequence of SseI is similar to several hypothetical proteins, two of which are from pathogenic bacteria species that are able to cause disease in humans [37],[38]. Similarity was also found to PMT/ToxA, a *P. multocida* toxin that inhibits DC migration [41], and alignment of all these sequences revealed several conserved amino acids, including C178 (Fig. 7A). PMT/ToxA was recently shown to be a deamidase that acts on heterotrimeric G proteins [40], and its activity as a toxin is dependent on a catalytic triad formed by the conserved residues, C1165, H1205, and D1220 (Fig. 7A) [49],[50]. We demonstrated that the substitution of C178 for an A in SseI impairs the ability of *S. typhimurium* to colonize host systemic sites and to inhibit directed host cell migration (Fig. 7B, 5A and 5B). However, these results also indicate that while IQGAP1 is required for SseI function in the host, binding of SseI to IQGAP1 is not sufficient because SseIC178A also binds IQGAP1 (Fig. 2C and S4). Thus, part of SseI-function also must be attributed to a specific activity that is dependent on C178. However, due to the fact that the conserved H216 and D231 were not essential for SseI-function (Fig. 5A), it is less apparent what this activity might be. Although a structural role for C178 cannot be ruled out, we have shown that the SseIC178A mutant protein is efficiently translocated into host cells and is stable (Fig. 7C and S4). Therefore, we hypothesize that SseI possesses a distinct biochemical activity that could act on IQGAP1. An alternative hypothesis is that SseI could be taking advantage of IQGAP1's role as a scaffolding protein [51] in order to be brought into contact with other host cell proteins (e.g. heterotrimeric G proteins, similar to PMT) that are altered by SseI, leading to a disruption in normal host cell migration. Characterization of SseI's associated biochemical activity is under active investigation.

A previous report by Worley et al. showed that SseI (SrfH) stimulated macrophage movement through and detachment from transwells and caused early escape of *S. typhimurium* from the GI tract into the blood stream [26]. This SseI-dependent activity also was shown to be dependent on the host protein, TRIP6, a factor required for normal cell adhesion [52]. Our results show that in RAW264.7 cells, SseI specifically regulates cell adherence (Fig. 6B), whereas in primary BMDM and BMDC, SseI blocks cell migration (Fig. 5A–5C). Taken together, these results provide evidence that SseI plays at least two different roles, one of which is to regulate cell adherence in order to cause early escape of *S. typhimurium* out of the GI tract and into the blood stream as reported previously [8],[26]. This role could explain the slight attenuation of the *ΔsseI* mutant at 3d in the liver, a highly perfused organ that also filters blood from the GI tract (Fig. 1C). However, our results clearly demonstrate that SseI also plays an important inhibitory role in the regulation of host cell migration. This role becomes critical during later stages of infection and allows *S. typhimurium* to maintain a long-term systemic infection of the host, as demonstrated by the striking increase in attenuation of the *ΔsseI* mutant that occurs between 30d and 45d post-infection in the spleen and liver (Fig. 1B and 1C).

We have demonstrated an SseI-dependent decrease of DC migration in vivo (Fig. 8A) which correlates with a decrease in DC and CD4^+^ T cell numbers in the spleens of mice infected with WT *S. typhimurium* (Fig. 8C and 8D). Previous reports have demonstrated that infection with virulent *Salmonella* strains correlated with reduced T cell activation [13],[15],[53] and identified SPI2-dependent suppression of DC-mediated antigen presentation as an underlying mechanism. However, SseI did not reduce the capacity of DC to stimulate T cell proliferation in vitro (Fig. S5B, S5C, S5D). Others have shown that specifically interfering with DC migration attenuates T cell proliferation in vivo [54]. Thus, a possible hypothesis is that SseI indirectly controls CD4^+^ T cell numbers by suppressing DC migration and limiting their ability to effectively prime naïve T cells. However, this global effect of *sseI* would not entirely account for the competitive advantage of WT bacteria over the *ΔsseI* mutant in mixed infections (Fig. 7B) and suggests that the SseI-mediated decrease in host cell migration also may reduce the accessibility of infected cells to local immune cell-mediated killing mechanisms. Nevertheless, the effects of SseI on host cell migration, through its interactions with the host molecule IQGAP1, correlate with a reduced capacity of the host to clear *S. typhimurium* from systemic sites of infection.

## Materials and Methods

### Ethics statement

All animal experiments were performed in accordance to NIH guidelines, the Animal Welfare Act, and US federal law. Such experiments were carried out under the supervision of Stanford University's Administrative Panel on Laboratory Animal Care (A-PLAC) which has been accredited by the Association of Assessment and Accreditation of Laboratory Animal Care International (AAALAC). All animals were housed in a centralized and AAALAC-accredited research animal facility that is fully staffed with trained husbandry, technical, and veterinary personnel.

### Mouse strains and mammalian cell culture

Female 129x1/sv J mice (6–8 weeks old) were obtained from Jackson Laboratories (Bar Harbor, ME). Bone marrow was harvested from femurs of *IQGAP1^−/−^* mice and WT littermate controls [55]. Marrow was differentiated into primary macrophages (BMDM) or dendritic cells (BMDC) as described previously [56],[57]. RAW264.7 cells were cultured in DMEM with 10% heat-inactivated FBS. Cell cultures were incubated in a humidified chamber at 37°C in 5% CO_2_.

### Bacterial strains and plasmid constructs

*S. typhimurium* SL1344 (2) was used as the parent strain for all experiments presented here, and the *ΔsseI* strain was created by replacing the *sseI* coding sequence with that of a kanamycin-resistance gene [58]. To complement, the *sseI* gene plus the 476 bp upstream sequence was cloned into the low copy number plasmid p*ACYC184* (p*sseI*), which also contains a chloramphenicol-resistance marker. The C178A mutation was generated by site-directed mutagenesis via the QuickChange II mutagenesis kit (Stratagene, La Jolla, CA) and cloned into p*ACYC184* (p*sseIC178A*). All recombinant protein expression constructs were generated using the Gateway cloning system (Invitrogen, Carlsbad, CA). *sseI* and *sseIC178A* were cloned into either pDEST15 to generate the N-terminal GST tag (p*GST-sseI* and p*GST-sseIC178A*) or pDEST17 to generate the N-terminal 6xHis tag (p*His-sseI* and p*His-sseIC178A*). p*HispipB* was also derived from pDEST17 and was a generous gift from Dr. Stephane Meresse (CIML Université de la Méditerranée, Marseille, France). Recombinant proteins were purified from *Escherichia coli* BL21 strain. The *sseI* gene was also cloned into pEGFP (modified for use with the Gateway system) to form p*sseI-GFP* and used for transient transfections in BMDM.

### Mouse infections

For competitive infection assays, 129x1/sv J mice (6–8 weeks old) were co-infected with equal amounts of the WT and mutant strains by IP injection (10^4^/strain, “input”). Homogenized tissues were plated on 200µg/ml streptomycin LB plates and on plates with both streptomycin and either 40µg/ml kanamycin or 8µg/ml chloramphenicol. The competitive index (CI) was calculated as (cfu mutant_output_/cfu WT_output_)/(cfu mutant_input_/cfu WT_input_). In single infections, mice were infected by IP (10^4^ cfu/mouse) or by oral gavage (10^8^ cfu/mouse).

### Flow cytometry

Single cell suspensions of spleens from naïve mice and mice infected with WT or *ΔsseI* mutant strains (IP) were prepared in RPMI, and the red blood cells were lysed in 175mM ammonium chloride, 10mM phosphate buffer, pH 7. 2×10^6^ cells were stained per sample. Rat anti-mouse CD16/CD32 (BD Pharmingen, San Jose, CA) was added to block FcIII/IIR prior to staining with analytical antibodies. Dead cells were stained a using Live/Dead Fixable dead cell stain kit (Invitrogen, Carlsbad, CA). The samples were stained with the analytical antibodies against the following cell surface markers: B7.2 (CD86), CD3e (2C11), CD4, CD11b (BD Pharmingen); CD11c, CD19, MHC-II (M5/114.15.2), TCRβ (H57) (eBioscience, San Diego, CA). In in vitro experiments, BMDC were stained with CD11c, MHC-II, and CCR7 (eBioscience, San Diego, CA) antibodies. Data were collected either on a LSR II (BD Biosciences) or on a modified FACStar (FlasherII, Diva Digital) at the Stanford University shared FACS facility, and the data were analyzed with FlowJo software (Treestar, Ashland, OR). DC were defined as MHC-II^+^ and CD11c^+^, and CD4^+^ T cells were defined as CD3^+^, TCRβ^+^ and CD4^+^. Absolute numbers of cells were calculated per g of spleen. Antigen presentation was assayed as described [15]. BMDC were infected with *S. typhimurium* for 2h and incubated with 10 µg/ml of pigeon cytochrome C. CFSE-labeled T cells purified from 5C.C7 transgenic mice (T cell receptor for I-Ek/moth cytochrome C_88–103_ [59]) were added to the BMDC in a 1∶1 ratio. At 3d, T cell-CFSE labeling was analyzed by flow cytometry.

### SseI protein purification and SseI-IQGAP1 in vitro and in vivo binding

SseI fusion proteins were purified using standard methods utilizing GSH conjugated- or nickel-charged resin, and purity was checked by SDS-PAGE and Coomassie stain [60]. Briefly, overnight cultures of *E. coli* BL21 transformed with p*His-sseI*, p*His-sseIC178A*, p*HispipB*, p*GST-sseI* or p*GST-sseIC178A* were diluted 50× and grown at 37°C with shaking until reaching an optical density between 0.7–0.9 at 600 nm. Cultures were heat-shocked at 42°C for 10 min, cooled to room temperature, and incubated with 1 mM IPTG for 1d. Cells were lysed by resuspending in BugBuster lysis buffer (EMD Chemicals, Inc., Gibbstown, NJ) with 50 mM AEBSF using a needle and syringe, and the lysate was cleared by spinning at 20,000g for 15 min at 4°C. The lysate was then combined with either GSH-agarose resin (incubated overnight at 4°C, for GST-tagged proteins), nickel-charged resin (incubated for 5 min at room temperature, for His-tagged proteins), or protein G plus resin (incubated for 3h at 4°C, EMD Chemicals Inc., Gibbstown, NJ). In the case of protein G plus resin, the lysate was first pre-incubated with anti-His tag antibody (R&D Systems, Minneapolis, MN) for 30 min on ice. The resins were then washed: 5 times in PBS for GSH-agarose resin, 3 times with 1× binding buffer (20 mM Tris-HCl pH 7.9, 500 mM NaCl, 5 mM imidazole) and 3 times with 1× wash buffer (20 mM Tris-HCl pH 7.9, 500 mM NaCl, 60 mM imidazole) for nickel-charged resin, or twice with lysis/binding buffer (1% NP-40, 150 mM NaCl, 50 mM TrisHCl pH 7.2, 2 mM EDTA, Na Vanadate 400 µM, 50 µM NaF and 1mM AEBSF) for protein G resin. The resin-bound SseI proteins were then used for in vitro binding assays, or nickel-charged resin-bound His-SseI proteins were eluted using 20 mM Tris-HCl pH 7.9, 500 mM NaCl, 1 M imidazole. Free His-SseI proteins were then dialysed against a buffer containing 20 mM Tris-HCl pH 7.9, 100 mM NaCl, and 0.3mM DTT before being used for in vitro binding assays as well. Purified GST-IQGAP1 was prepared as previously described by Ho et al. [61].

Approximately 5×10^6^ BMDM or BMDC were lysed in 800 µl lysis/binding buffer. Cleared cell lysates were pre-incubated with GSH-resin for 2h at 4°C before combining with a given GST fusion protein pre-bound to GSH-resin and incubated overnight at 4°C. The resin was washed, and bound proteins were eluted by boiling in 1× SDS-sample buffer: 125 mM Tris-HCl pH 6.8, 1.8% SDS, 5% glycerol, 0.1 M dithiothreitol, and 0.002% bromphenol blue. Free IQGAP1 was generated by cleaving the GST tag with His-tagged Tobacco Etch Virus (TEV) protease and combined with resin-bound His-SseI (or His-PipB) in lysis/binding buffer for overnight incubation at 4°C, or resin-bound GST-IQGAP1 was incubated with free His-SseI and incubated overnight at 4°C. The resin was washed 5 times with lysis/binding buffer and bound proteins were eluted as before. All eluates were subjected to SDS-PAGE and Coomassie staining or immunoblotting. The membranes were stained with antibodies reactive against IQGAP1 (Santa Cruz Biotechnology, Santa Cruz, Ca), 6X-His tag (R&D Systems, Minneapolis, Mn), rabbit IgG-660 (Molecular Probes, Carlsbad, CA), and mouse IgG-800 (Rockland Immunochemicals, Gilbertsville, PA) and detected using the Odyssey system (Li-Cor Biosciences, Lincoln, NE).

To detect SseI-IQGAP1 binding in the context of an infection, BMDM (caspase-1^−/−^) were infected (MOI of 25) with *S. typhimurium* (with or without p*sseI-cya*) grown standing in Luria Broth to induce SPI1 expression. At 6h, harvested proteins were subjected to co-immunoprecipitation with anti-CyaA antibody (Santa Cruz Biotechnology, Santa Cruz, Ca) and immunoblotted for IQGAP1 or TRIP6 (antibody was a generous gift from Dr. M. Beckerle, University of Utah).

### Transient transfections and fluorescence microscopy

5×10^6^ BMDM were combined with 5 µg of p*sseI-GFP* or p*EGFP* and electroporated using the Amaxa Nucleofector device (Lonza, Cologne, Germany). Immediately afterward, BMDM were seeded onto coverslips and later fixed in 2% *para*-formaldehyde phosphate buffer. BMDM were stained with anti-IQGAP1 antibody (1∶50, Santa Cruz, CA) and Alexa fluor 647 phalloidin (1∶50, Molecular Probes, Carlsbad, CA), and z-stack images were taken by confocal microscopy at 600× and analyzed using Volocity software (Improvision Inc., Waltham, MA).

### BMDM and BMDC in vitro and in vivo migration and motility assays

One day prior to infection, BMDM or BMDC were seeded onto transwell inserts for 24-well plates (5 µm pore size, Corning, Corning, NY) at 1×10^5^ or 2.5×10^5^ cells/well, respectively. *Salmonella* strains were opsonized in a 1∶1 solution of normal mouse serum and cellular medium solution, and then used to infect BMDM or BMDC at a multiplicity of infection (MOI) of 10∶1. Extracellular bacteria were killed by adding 100 µg/ml gentamicin after 30 minutes, and 1.5h later reduced to 10 µg/ml gentamicin. Each infection was done in duplicate wells. An attractant was added to the bottom chamber of one well from each infection at 24h. An equivalent of 12.5 million cfu of heat-killed *Salmonella* (WT, boiled 10min in PBS) was used as the attractant for BMDM, and 100ng/ml CCL19 (PeproTech, Rocky Hill, NJ) was used for BMDC. Five hours later, the cells were fixed to the membrane, stained for nuclei using DAPI, and the percentage of cells migrating to the bottom side of the filter was counted by confocal microscopy (at least 300 cells were counted per sample). The percent directed migration was reported as the difference: (% migration toward the added attractant) – (% migration without added attractant). For migration assays independent of *S. typhimurium* infection, murine macrophages (WT and IQGAP1^−/−^) seeded on to transwell filters (8 µm pore size, coated on the underside with fibronectin) were placed over chambers, each containing 600 µl medium with macrophage-colony stimulating factor (100 ng/ml). After 5h of incubation at 37°C, the migrated cells attached to the bottom surface of the transwell filters were stained with Diff-Quick, and the numbers of migrated cells per filter were counted in 10 random fields with an inverted microscope.

In vivo DC migration was measured by staining BMDC with PKH26, and then infecting these cells with *S. typhimurium* SL1344 strains (WT or *ΔsseI*) transformed with the GFP expression vector p*FPV25.1* [62]. Infections were carried out as above, except with an MOI of 50∶1 such that 49±1.6 % of the cells were infected. Infected cells were incubated in 100µg/ml gentamicin to kill extracellular bacteria, washed and resuspended in PBS at 25 million cells/ml. 129x1/sv J mice were injected IP with 0.2ml of this suspension. Six hours later, the mice were sacrificed and single cell suspensions of the spleens were prepared and immediately subjected to FACS analysis (BD LSR II) to quantify the number of PKH26^+^/GFP^−^ and PKH26^+^/GFP^+^ cells that had successfully migrated to the spleen (“output”). Approximately 40% of the total number of injected PKH26^+^ BMDC migrated to the spleen by 6h post-injection, and experiments comparing mock-infected BMDC and *Salmonella*-infected BMDC confirmed that *Salmonella* infection did not significantly affect the total numbers of PKH26^+^ (GFP^+^ and GFP^−^) cells found in the spleen. At the same time, the original PKH26-stained infected BMDC (used for mouse injections) were analyzed by FACS (“input”). The data were analyzed using FlowJo, and the results are reported as the in vivo migration index = [[(#PKH26^+^GFP^+^ cells)/(#PKH26^+^GFP^−^ cells)]_output_]/[[(#PKH26^+^GFP^+^ cells)/(#PKH26^+^GFP^−^ cells)]_input_]. This in vivo migration index allowed us to control for any slight differences in the amount of BMDC injected between mice.

Time-lapse microscopy was carried out using a Nikon TE2000E microscopy (inside a controlled chamber held at 37°C and 5% CO_2_) and images recorded using a Hamamatsu Electron Multiplier C9100-12 back-thinned CCD camera and processed using Openlab software (Improvision, Waltham, MA). Briefly, BMDM were seeded onto 2-chamber glass slides (100,000 cells/chamber) and cells were infected with either WT(p*FPV25.1*) or *ΔsseI*(p*FPV25.1*) *S. typhimurium* SL1344 expressing GFP (as above, but with MOI of 100∶1). After incubating for 20h, 2 to 4 points in each chamber were chosen for time-lapse microscopy (points were imaged 45 times with a 3 min lapse between each imaging). Both DIC and green fluorescence were detected at 400× magnification, and the corresponding videos were compiled into Quicktime (Apple, Cupertino, CA) movies. The cells were tracked by their nuclei using ImageJ (http://rsbweb.nih.gov/ij/index.html), and the tracks were analyzed using Excel (Microsoft, Seattle, WA). The results are presented as the number of times a given cell changes its direction of travel more than 90° per 45 frame movie, and the angle of direction-change was calculated by taking the arccosine of the dot product of the vectors formed by a given cell's positions in 3 consecutive frames ((x_1_, y_1_), (x_2_, y_2_), and (x_3_, y_3_)) divided by the product of the magnitude of these vectors. Thus, the angle of direction-change is equal to the arccosine[[(x_2_−x_1_)*(x_3_−x_2_)+(y_2_−y_1_)*(y_3_−y_2_)]/[(((x_2_−x_1_)^2^+(y_2_−y_1_)^2^)^0.5^)* (((x_3_−x_2_)^2^+(y_3_−y_2_)^2^)^0.5^)]], and the radians converted into degrees by multiplying by 180/π. The results from each treatment group were compiled from 14 separate videos that were done on 4 different days.

### *Salmonella* infection and detachment assays

The efficiency of bacterial uptake and survival inside of BMDM was determined as previously described by Brodsky et al. (2005). Briefly, WT and IQGAP1^−/−^ BMDM seeded in 24-well plates (2.5×10^5^ cells/well) were infected with opsonized WT *S. typhimurium* (as in the migration assay). The infected BMDM were lysed 2h and 24h after initiating the infection and plated for cfu. The number of intracellular bacteria was recorded as a percent of the input, and each experiment was performed in triplicate.

To measure the loss of cell adherence of infected macrophages, BMDM and RAW264.7 cells were seeded into 6-well plates (5×10^5^ cells/well) and infected at an MOI of 10∶1 as in the cell migration assay (2 wells/replicate, 3 replicates per sample). Immediately after changing the cell medium to 10µg/ml gentimicin, heat-killed *Salmonella* was added to one half of the wells, and 24h later detached cells were harvested, lysed gently in 1% triton-X100, and plated for cfu. The cfu recovered from detached cells was recorded as the number of cfu in detached cells per well, and the results are reported as the difference: (# cfu in detached cells treated with heat-killed *Salmonella*) – (# cfu in detached cells without heat-killed *Salmonella*).

### Adenylate cyclase assay

The coding sequence of the first 399 residues of CyaA (adenylate cyclase domain) from *B. pertussis* was cloned in frame onto the 3′ end of *sseI* constructs (omitting the *sseI* stop codon), creating p*sseI*-*cya* and p*sseIC178A*-*cya*. WT and *ΔssaV* (SPI2 T3SS deficient) *S. typhimurium* strains were transformed with p*sseI-cya* or p*sseIC178A-cya* and used to infect RAW264.7 cells seeded in 6-well plates. Infected cells were lysed 6h later by sonication (10 sec at 40%, 6 times, at 4°C) and cleared by centrifugation. After reserving an aliquot of the lysate for protein determination by Bradford assay (Bio-Rad, Hercules, CA), the following reaction buffer was added to the lysates (final concentration: 2mM ATP, 6mM MgCl_2_, 100µg/mL bovine serum albumin, 0.12 mM CaCl_2_, and 0.1 µM calmodulin) and immediately assayed for cAMP content using the cAMP EIA kit from Cayman Chemical (Ann Arbor, Mi).

### Sequence alignment and statistics

A position iterative (PSI) algorithm of the Blast search engine (http://www.ncbi.nlm.nih.gov/blast/Blast.cgi) was used with default BLOSUM62 parameters and a 0.005 threshold to search the non-redundant protein database for homology to SseI (159–322 aa). Hypothetical proteins from *B. dolosa* and *P. asymbiotica* were identified by scanning unfinished microbial genome databases at NCBI and Sanger Center, respectively. The alignment was prepared using VectorNTI 9 (Invitrogen, Carlsbad, CA) and GeneDoc (ref at http://www.nrbsc.org/gfx/genedoc/gdfeedb.htm). Error bars represent standard errors of the mean, all results are representative of at least 3 experiments (unless stated otherwise), and all statistics were calculated using either Microsoft Excel or Graphpad Prism 4.

## Supporting Information

Figure S1 SseI is not required for intracellular bacterial survival or for *S. typhimurium*-induced host cell death. A and B) WT BMDM (A) or RAW264.7 macrophages (B) were infected with WT (black squares), *ΔsseI* (grey triangles), or *ΔsseJ* (white upside down triangles) strains of *S. typhimurium*, and the amount of intracellular bacteria was measured by plating for cfu at the indicated times. C and D) BMDC were infected as in Fig. 4B with the indicated strains (C) or with these strains grown under SPI1-inducing conditions (D). Host cell death was measured at 24h (C) or 6h (D) by measuring the leakage of lactate dehydrogenase (LDH).
(0.01 MB PDF)

Figure S2 Analysis of SseI and IQGAP1 co-localization. A and B) These images were taken directly from Fig. 3A and 3B, respectively; green staining denotes SseI-GFP (A) or GFP (B) and red staining denotes endogenous IQGAP1. Lines were drawn through the lamella and the red and green pixel intensities were measured along these lines from top to bottom using ImageJ. C and D) The plot profiles from each image are shown on the right; green line represents GFP intensities and red line represents IQGAP1-staining intensities (SseI-GFP, C; GFP, D).
(0.57 MB PDF)

Figure S3 *IQGAP1^−/−^* BMDM are less motile than WT BMDM. WT and *IQGAP1^−/−^* murine macrophages were similarly seeded onto transwells and M-CSF (100 ng/ml) was added to the baso-lateral compartment for 5h. The number of cells that migrated through the filter was counted (cells/field) and is presented as the percent of WT. Ten fields were counted per sample, and the results are presented as the average ± standard deviation of two independent experiments.
(0.00 MB PDF)

Figure S4 SseI and SseIC178A proteins both can bind IQGAPl. A) Increasing amounts of *E. coli* extracts over-expressing His-tagged SseI proteins (WT and C178A) were incubated with GST or GST-IQGAP1 and then co-precipitated with GSH-agarose resin. Bound proteins were immunoblotted using anti-His antibody as in Fig. 2C.
(0.02 MB PDF)

Figure S5 SseI-regulation of cellular composition of the spleen in vivo, DC-mediated T cell proliferation in vitro, and DC surface marker expression. A) The spleens mice were infected as described in Fig. 8C and 8D, and the number of GR-1^+^ cells were determined. B–D) The effect of BMDC infected with WT (C), *ΔssaV* (D), or *ΔsseI* (E) *S. typhimurium* on T cell proliferation was measured by co-culturing 5cc7 (moth cytochrome C-reactive) T cells with the infected BMDC and 10µg/ml cytochrome C. To detect proliferation, T cells were pre-stained with Carboxyfluorescein succinimidyl ester (CFSE), and staining was measured by FACS. Each peak indicates one round of cell division; representative histograms are shown, n = 2. E and F) Surface expression of MHC-II (E) and B7.2 (F) on DC isolated from the spleens of infected mice was assessed by staining with specific antibodies. G and H) BMDC were infected with the indicated strains of *S. typhimurium* (UVK = ultraviolet radiation-killed *S. typhimurium*), and one day later, the cell surface expression of CCR7 (G) and MHC-II (H) was analyzed by flow cytometry. Representative histograms are shown.
(0.56 MB PDF)

Video S1 Time-lapse video microscopy, WT *S. typhimurium*-infected BMDM.
(2.28 MB MOV)

Video S2 Time-lapse video microscopy, *ΔsseI S. typhimurium*-infected BMDM.
(1.73 MB MOV)
