# Supplementary figures and images for: The Salmonella SPI2 Effector SseI Mediates Long-Term Systemic Infection by Modulating Host Cell Migration
Source: PLoS Pathog. 2009 Nov 26;5(11):e1000671. doi: 10.1371/journal.ppat.1000671 (PMC2777311; doi:10.1371/journal.ppat.1000671)

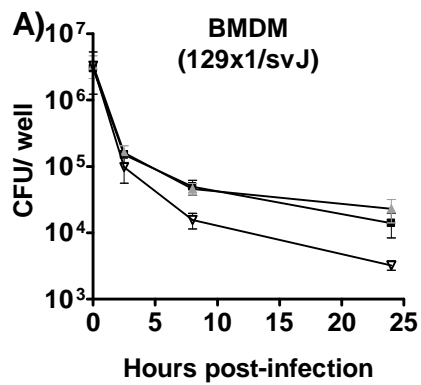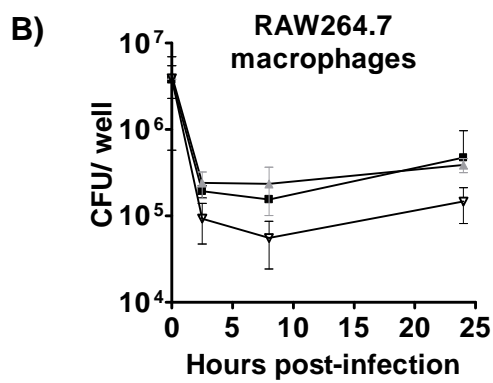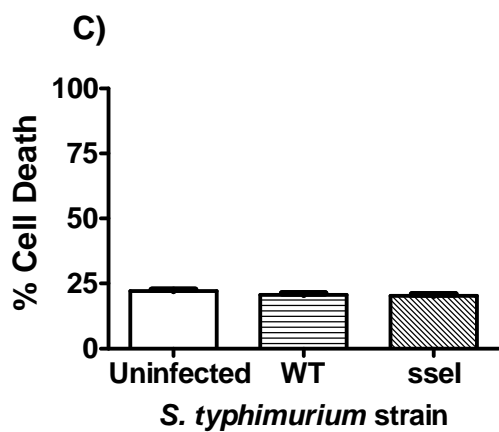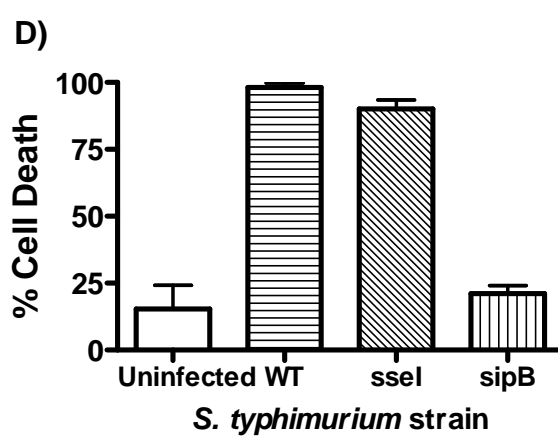

Supplement: Figure S1 — SseI is not required for intracellular bacterial survival or for S. typhimurium-induced host cell death. A and B) WT BMDM (A) or RAW264.7 macrophages (B) were infected with WT (black squares), ΔsseI (grey triangles), or ΔsseJ (white upside down triangles) strains of S. typhimurium, and the amount of intracellular bacteria was measured by plating for cfu at the indicated times. C and D) BMDC were infected as in Fig. 4B with the indicated strains (C) or with these strains grown under SPI1-inducing conditions (D). Host cell death was measured at 24h (C) or 6h (D) by measuring the leakage of lactate dehydrogenase (LDH). (0.01 MB PDF) [file ppat.1000671.s001.pdf]

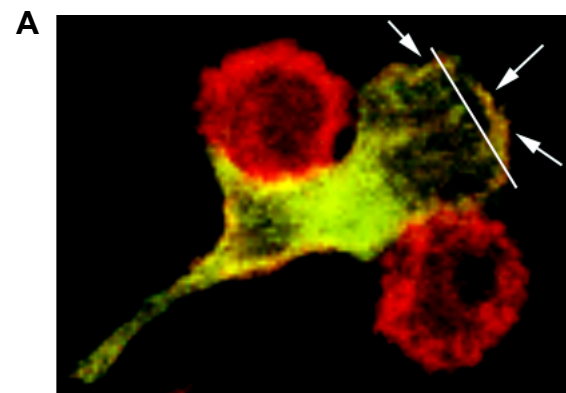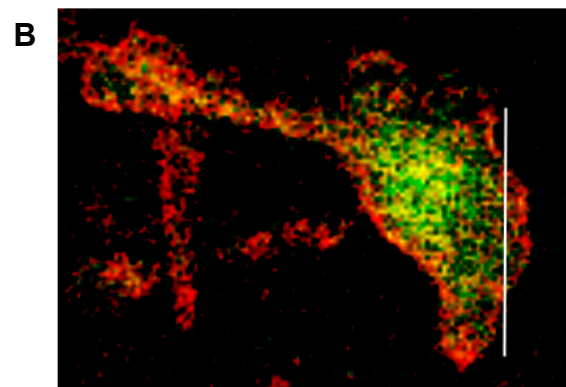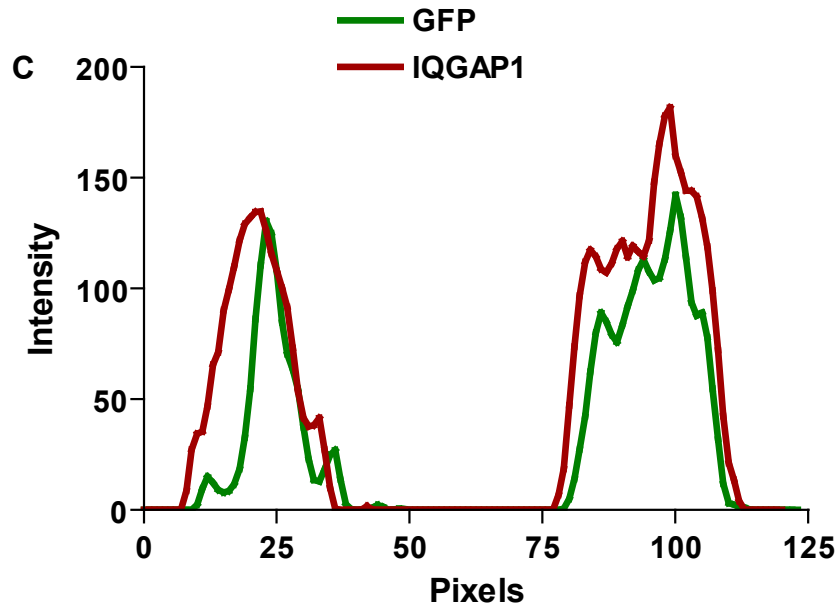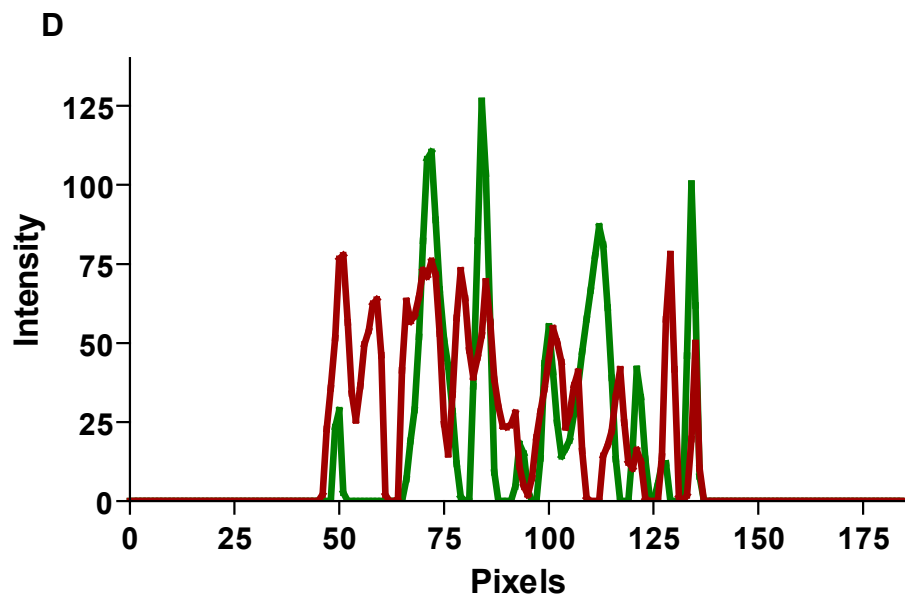

Supplement: Figure S2 — Analysis of SseI and IQGAP1 co-localization. A and B) These images were taken directly from Fig. 3A and 3B, respectively; green staining denotes SseI-GFP (A) or GFP (B) and red staining denotes endogenous IQGAP1. Lines were drawn through the lamella and the red and green pixel intensities were measured along these lines from top to bottom using ImageJ. C and D) The plot profiles from each image are shown on the right; green line represents GFP intensities and red line represents IQGAP1-staining intensities (SseI-GFP, C; GFP, D). (0.57 MB PDF) [file ppat.1000671.s002.pdf]

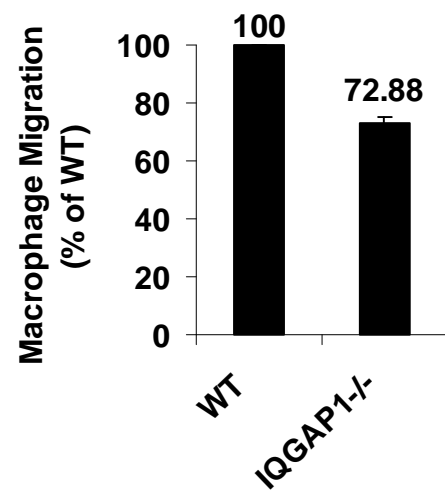

Supplement: Figure S3 — IQGAP1−/− BMDM are less motile than WT BMDM. WT and IQGAP1−/− murine macrophages were similarly seeded onto transwells and M-CSF (100 ng/ml) was added to the baso-lateral compartment for 5h. The number of cells that migrated through the filter was counted (cells/field) and is presented as the percent of WT. Ten fields were counted per sample, and the results are presented as the average ± standard deviation of two independent experiments. (0.00 MB PDF) [file ppat.1000671.s003.pdf]

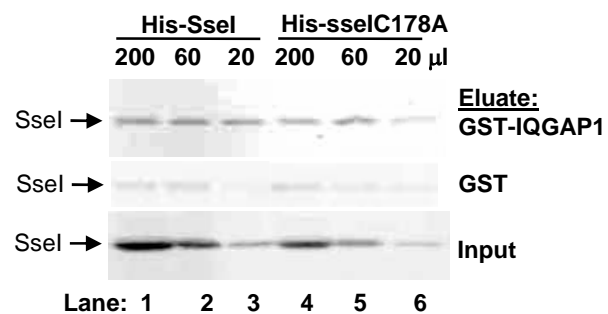

Supplement: Figure S4 — SseI and SseIC178A proteins both can bind IQGAPl. A) Increasing amounts of E. coli extracts over-expressing His-tagged SseI proteins (WT and C178A) were incubated with GST or GST-IQGAP1 and then co-precipitated with GSH-agarose resin. Bound proteins were immunoblotted using anti-His antibody as in Fig. 2C. (0.02 MB PDF) [file ppat.1000671.s004.pdf]

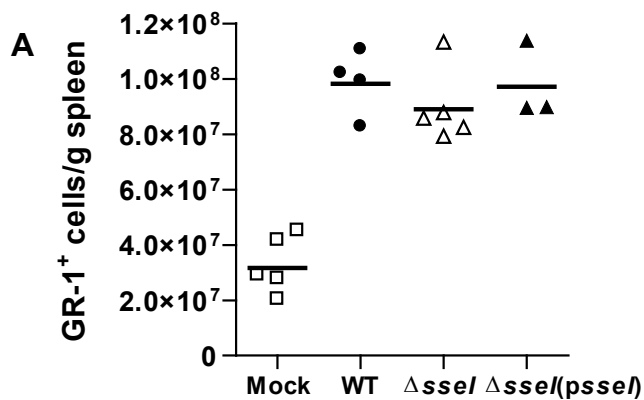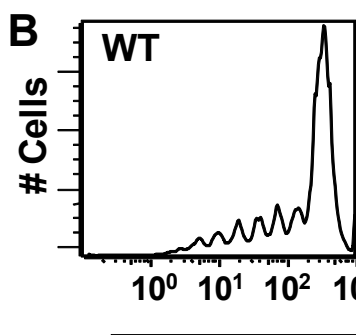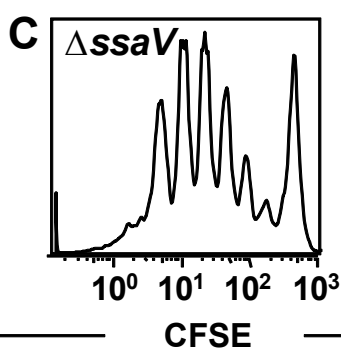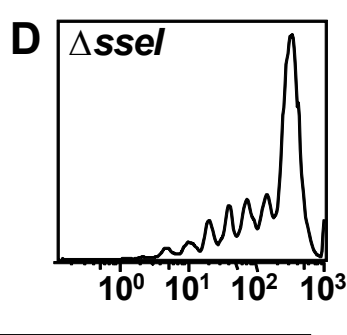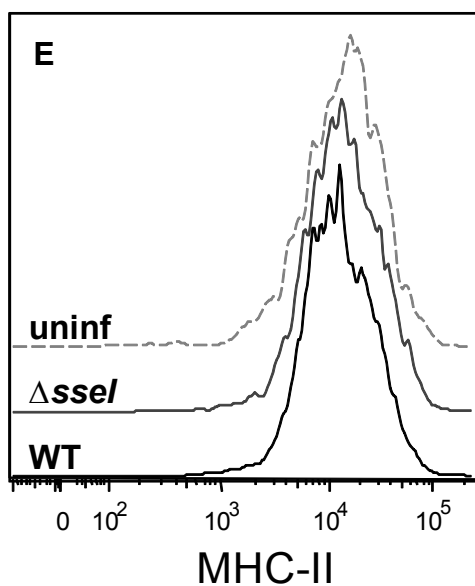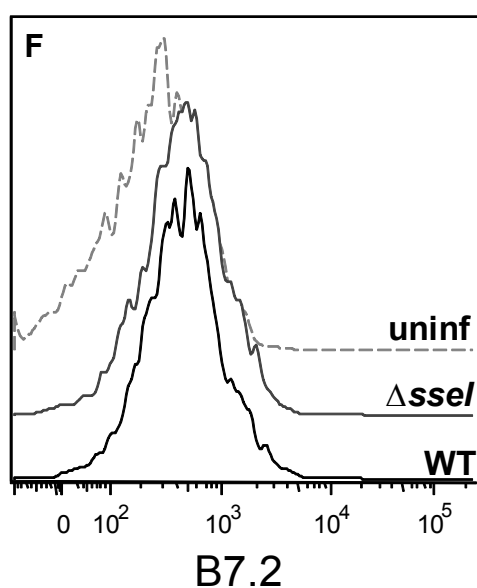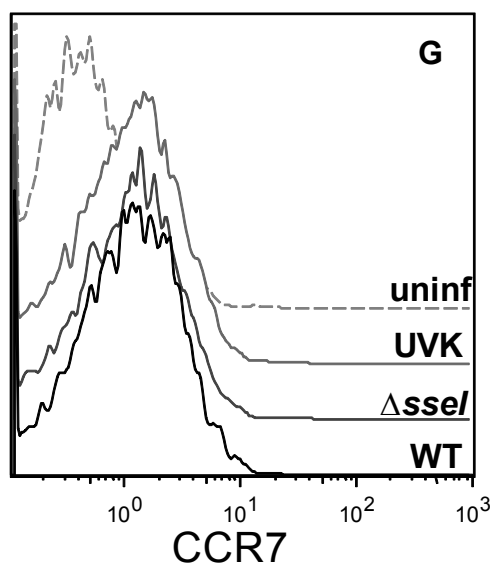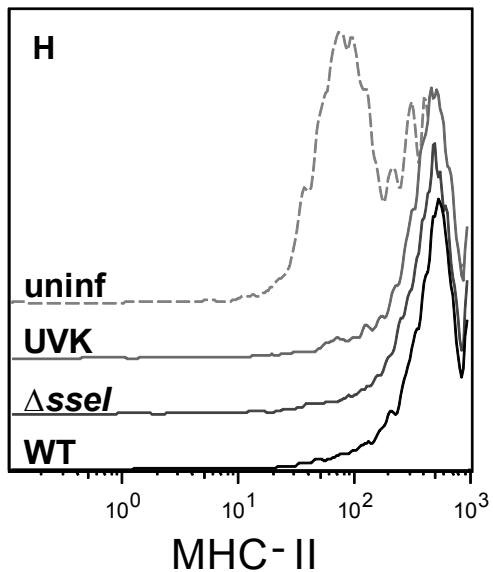

Supplement: Figure S5 — SseI-regulation of cellular composition of the spleen in vivo, DC-mediated T cell proliferation in vitro, and DC surface marker expression. A) The spleens mice were infected as described in Fig. 8C and 8D, and the number of GR-1+ cells were determined. B–D) The effect of BMDC infected with WT (C), ΔssaV (D), or ΔsseI (E) S. typhimurium on T cell proliferation was measured by co-culturing 5cc7 (moth cytochrome C-reactive) T cells with the infected BMDC and 10µg/ml cytochrome C. To detect proliferation, T cells were pre-stained with Carboxyfluorescein succinimidyl ester (CFSE), and staining was measured by FACS. Each peak indicates one round of cell division; representative histograms are shown, n = 2. E and F) Surface expression of MHC-II (E) and B7.2 (F) on DC isolated from the spleens of infected mice was assessed by staining with specific antibodies. G and H) BMDC were infected with the indicated strains of S. typhimurium (UVK = ultraviolet radiation-killed S. typhimurium), and one day later, the cell surface expression of CCR7 (G) and MHC-II (H) was analyzed by flow cytometry. Representative histograms are shown. (0.56 MB PDF) [file ppat.1000671.s005.pdf]
